# Auxin‐induced actin cytoskeleton rearrangements require AUX1

**DOI:** 10.1111/nph.16382

**Published:** 2020-02-11

**Authors:** Ruthie S. Arieti, Christopher J. Staiger

**Affiliations:** ^1^ Department of Biological Sciences Purdue University West Lafayette IN 47907‐2064 USA; ^2^ Purdue University Interdisciplinary Life Sciences Graduate Program (PULSe) Purdue University West Lafayette IN 47907 USA; ^3^ Center for Plant Biology Purdue University West Lafayette IN 47907 USA; ^4^ Department of Botany and Plant Pathology Purdue University West Lafayette IN 47907 USA

**Keywords:** actin, Arabidopsis, AUX1, auxin, cell expansion, cytoskeleton, signaling

## Abstract

- The actin cytoskeleton is required for cell expansion and implicated in cellular responses to the phytohormone auxin. However, the mechanisms that coordinate auxin signaling, cytoskeletal remodeling and cell expansion are poorly understood. Previous studies examined long‐term actin cytoskeleton responses to auxin, but plants respond to auxin within minutes. Before this work, an extracellular auxin receptor – rather than the auxin transporter AUXIN RESISTANT 1 (AUX1) – was considered to precede auxin‐induced cytoskeleton reorganization.
- In order to correlate actin array organization and dynamics with degree of cell expansion, quantitative imaging tools established baseline actin organization and illuminated individual filament behaviors in root epidermal cells under control conditions and after indole‐3‐acetic acid (IAA) application. We evaluated *aux1* mutant actin organization responses to IAA and the membrane‐permeable auxin 1‐naphthylacetic acid (NAA).
- Cell length predicted actin organization and dynamics in control roots; short‐term IAA treatments stimulated denser and more parallel, longitudinal arrays by inducing filament unbundling within minutes. Although AUX1 is necessary for full actin rearrangements in response to auxin, cytoplasmic auxin (i.e. NAA) stimulated a lesser response.
- Actin filaments became more ‘organized’ after IAA stopped elongation, refuting the hypothesis that ‘more organized’ actin arrays universally correlate with rapid growth. Short‐term actin cytoskeleton response to auxin requires AUX1 and/or cytoplasmic auxin.

## Introduction

Despite human dependence on plants, we do not fully understand the molecular mechanisms controlling plant growth. Many plant cells expand isotropically but, during development, establish polar growth where deposition of cell wall materials is restricted to specific cell axes, producing mature cells with myriad shapes and sizes. Turgor pressure drives expansion within the confines of cell wall flexibility: plant cell wall flexibility is not uniform; looser regions are more susceptible to turgor pressure (Szymanski & Cosgrove, 2009; Guerriero *et al.*, 2014). Vesicles are incorporated into certain areas of the plasma membrane (PM) and deposit new cell wall material, increasing cell surface area and conducting cell growth into specific shapes. Vesicle delivery and exocytosis depend on the actin cytoskeleton, a dynamic array of filamentous structures in the cytoplasm of cells (Ketelaar *et al.*, 2003; Hussey *et al.*, 2006; Leucci *et al.*, 2007; Zhang *et al.*, 2019). Although the actin cytoskeleton is required for plant cell expansion (Baluška *et al.*, 2001; Gilliland *et al.*, 2003; Mathur, 2004; Hussey *et al.*, 2006; Rahman *et al.*, 2007; Kandasamy *et al.*, 2009; Yang *et al.*, 2011; Guerriero *et al.*, 2014), actin's function in this process is not well understood. Actin is accepted to provide tracks for vesicle delivery (Mathur, 2004; Hussey *et al.*, 2006), but connections also have been made between certain actin arrays and plant growth (Nick *et al.*, 2009; Higaki *et al.*, 2010a; Smertenko *et al.*, 2010; Dyachok *et al.*, 2011; Yang *et al.*, 2011, Yanagisawa *et al.*, 2015), resulting in various hypotheses about actin's role and/or the significance of specific actin arrays, each with supporting evidence, much of it circumstantial (Li *et al.*, 2015a; Szymanski & Staiger, 2017).

Actin arrays form an apparently ‘organized’ orientation, with actin bundles roughly parallel to the cell's longitudinal axis in rapidly growing root epidermal cells in the light (Dyachok *et al.*, 2011). In the dark, where cell expansion slows substantially, actin exhibits apparent ‘disorganization’: filaments are less aligned to the longitudinal axis of root cells (Dyachok *et al.*, 2011). However, the literature does not present data substantiating cause‐and‐effect. Whether a longitudinal array is necessary for, coincides with, promotes, or (conversely) is the product of, cell expansion – or whether the ‘disorganized’ array inhibits or coincides with a cessation of expansion – is not understood and remains largely unexamined.

In addition to longitudinal orientation, other actin arrays have been correlated with cell length or expansion. However, there is no consensus on whether longitudinal bundles inhibit (Gilliland *et al.*, 2003; Holweg *et al.*, 2004; Rahman *et al.*, 2007) or stimulate (Kandasamy *et al.*, 2009; Yang *et al.*, 2011; G. Li *et al.*, 2014) axial cell expansion. Many previous studies linking specific actin organizations with growth or growth inhibition are based on actin or actin‐binding protein mutant phenotypes (Gilliland *et al.*, 2003, Kandasamy *et al.*, 2009, Yang *et al.*, 2011; G. Li *et al.*, 2014). Others are based on actin responses to drug or long‐term hormone treatments (Holweg *et al.*, 2004; Rahman *et al.*, 2007). Therefore, some actin–cell growth models may be generalized from potentially more discrete responses: cytoskeletal response to an external stimulus that affects growth via downstream mechanisms; or filament array changes due to a missing or deficient actin‐binding protein whose role could be only one of many aspects of growth.

What tasks, exactly, actin undertakes during cell expansion and how these tasks drive or participate in expansion are unclear. Bundles potentially inhibit growth by inhibiting transport of growth hormone‐related proteins (Nick, 2010). Alternatively, long actin bundles presumably stimulate growth because they provide tracks for vesicle delivery (Szymanski & Cosgrove, 2009; Thomas, 2012). Actin bundles could contribute to vacuolar osmotic pressure regulation by altering turgor pressure (Higaki *et al.*, 2010a,b, 2011), the main driver of plant cell expansion (Szymanski & Cosgrove, 2009). A recent paper shows that auxin, a known plant growth modulator with rapid, opposing effects on root or shoot growth (inhibition and stimulation, respectively), constricts vacuolar shape in long‐term treatments (6+ h) on root cells by inducing altered actin arrays (Scheuring *et al.*, 2016). Interactions between auxin signaling pathways and actin are abundant in the literature (reviewed in Zhu & Geisler, 2015), but the mechanics of how auxin affects the cytoskeleton within minutes, and how these interactions alter growth, are largely unknown.

The molecular players that connect the actin cytoskeleton to auxin perception during short‐term responses are unidentified. Auxin perception by AUXIN BINDING PROTEIN 1 (ABP1) was suspected to precede cytoskeletal changes in roots (Chen *et al.*, 2012; Lin *et al.*, 2012) and epidermal pavement cells (Xu *et al.*, 2010; Nagawa *et al.*, 2012; Xu *et al.*, 2014). However, recent works demonstrate that, like wild‐type (WT) plants, a CRISPR *abp1‐c1* mutant exhibited root growth inhibition by both the natural auxin indole‐3‐acetic acid (IAA) and the highly membrane‐permeable, lipophilic synthetic auxin, 1‐naphthylacetic acid (NAA; Delbarre *et al.*, 1996; Carrier *et al.*, 2008), indicating that ABP1 likely plays no role in auxin signaling (Dai *et al.*, 2015; Gao *et al.*, 2015). AUXIN RESISTANT 1 (AUX1) is a PM‐bound auxin/H^+^ symporter in the amino acid/auxin permease (AAAP) family that is ubiquitous among Eukaryotes. AUX1 appears in all plants and some algae, indicating that it evolved before land plants (reviewed in Swarup & Péret, 2012). Unlike WT, *aux1* plants grow in the presence of IAA but undergo growth inhibition by NAA (Marchant *et al.*, 1999); AUX1 binds both IAA and NAA with high affinity (Yang *et al.*, 2006; Carrier *et al.*, 2008) and is responsible for 80% of IAA uptake by root hairs (Dindas *et al.*, 2018). The loss of auxin uptake by *aux1* roots enables growth in the presence of moderate IAA doses, but NAA inhibits *aux1* growth within seconds, in a similar way to the WT (Fendrych *et al.*, 2018). AUX1 contributes to short‐term, auxin‐induced increases in cytosolic H^+^ and, with the intracellular auxin receptor complex SCF^TIR1/AFB^ (which perceives auxin molecules, Kepinski & Leyser, 2005), increases in cytosolic Ca^2+^ (Dindas *et al.*, 2018), ultimately inducing transcriptional reprogramming (Ulmasov *et al.*, 1999). For years, AUX1's contribution to auxin uptake was deemed insignificant: IAAH diffuses from the acidic apoplast through the PM, so intracellular auxin concentrations and polar auxin transport must be controlled solely by export (Zhu & Geisler, 2015). We wanted to revisit AUX1’s role in short‐term auxin signaling and determine whether cytoplasmic auxin plays a role in signaling to the actin cytoskeleton.

In order to correlate actin arrays with cell expansion, we used quantitative tools to establish a baseline of actin organization and individual filament behaviors in root epidermal cells under control circumstances. By plotting measurements of each cell's actin array against its length, we found that cell length was highly predictive of actin organization and dynamics. We then used acute IAA treatments to determine the actin response in presumed non‐ or very slow‐growing cells and documented the first short‐term actin responses to exogenous IAA. Upon analyzing the actin arrays in two *aux1* alleles (the T‐DNA insertion mutant *aux1‐100* and the null point mutant *aux1‐22*), we found that actin failed to reorganize in response to IAA and reorganization was only partially restored by NAA. Our data substantiate that AUX1 and cytosolic auxin play significant roles upstream of actin reorganization in auxin signaling.

## Materials and Methods

### Plant material and growth conditions

All experiments were conducted on roots from 6‐d‐old, light‐grown *Arabidopsis thaliana* seedlings expressing GFP‐fABD2 (green fluorescent protein fused to the second actin‐binding domain of Arabidopsis FIMBRIN1): Col‐0, Wassilewskija (WS), *aux1‐100* and *aux1‐22*. Seeds were surface‐sterilized and stratified at 4°C for 2 d. Unless specified otherwise, experiments were conducted using Col‐0; WS was used as the wild‐type (WT) for *aux1‐100* because the *aux1‐100* mutation is in the WS background. All plants were grown on ½ Murashige & Skoog (½MS) medium solidified with 0.6% (w/v) agar and no sucrose, as described previously (Sheahan *et al.*, 2004; Dyachok *et al.*, 2011; Henty *et al.*, 2011; Cai *et al.*, 2014; J. Li *et al.*, 2014). For the light sheet fluorescence microscopy (LSFM), plants were grown in capillary tubes in ½MS medium solidified with 0.6% (w/v) agar and no sucrose. Detailed methods for LSFM are in Supporting Information Methods S1. All seedlings were grown vertically at 21°C with a light intensity of 117 μM photons m^−2^ s^−1^, under long‐day conditions (16 h : 8 h, light : dark).

The T‐DNA insertion mutant *aux1‐100* (CS2360) and ethyl methanesulphonate (EMS) point mutant *aux1‐22* (CS9585) were obtained from the ABRC stock center and, with WS‐0 and Col‐0, transformed with GFP‐fABD2 (Sheahan *et al.*, 2004) using the floral dip method (Zhang *et al.*, 2006). T_1_ plants were screened on plates with hygromycin. PCR genotyping confirmed *aux1‐100* plant homozygosity; primers (Krysan *et al.*, 1996) appear in Methods S2. *aux1‐22* mutants were identified by their agravitropic phenotype. T_2_ plants were used for experiments.

### VAEM imaging, measuring cell lengths and quantitative analysis of cortical actin array organization

In order to measure cell sizes and obtain a corresponding measurement of each actin organization parameter, we collected overlapping variable angle epifluorescence microscopy (VAEM) images (single optical sections) of cortical cytoplasm from the outer periclinal face of root epidermal cells expressing GFP‐fABD2. Images were collected from the root elongation zone: root apex (i.e. root cap) to the first obviously visible root hair initiations (end of the elongation zone/beginning of the differentiation zone).

VAEM used a total internal reflection fluorescence (TIRF) illuminator mounted on an IX‐71 microscope equipped with a 60 × 1.45–numerical aperture PlanApo TIRF objective (Olympus Corp., Waltham, MA, USA). Illumination was from a solid‐state 50 mW laser (Intelligent Imaging Innovations, Denver, CO, USA) at 3–5% power and kept constant for a single experiment/replicate. An electron multiplying charge‐coupled device camera (ORCA‐EM C9100‐12; Hamamatsu Photonics, Hamamatsu, Japan) captured 488‐nm laser emission. The microscope platform was operated and images collected with slidebook software (v.6; Intelligent Imaging Innovations). Fixed exposure and gain were selected so individual actin filaments were visible but higher order filament structures were not intensity‐saturated. Detailed methods for actin organization analysis are in Methods S3.

### Auxin treatments

IAA (I2886) and NAA (N0640) were obtained from Sigma‐Aldrich and diluted to a 10 mM stock concentration in ultrapure ethanol (BP2818500; FisherScientific, Waltham, MA, USA). For experiments, each stock was diluted to appropriate concentrations into ½MS liquid medium without sucrose; for control solution, ultrapure ethanol was added to ½MS liquid medium without sucrose to match the highest auxin concentration used. Because light degrades auxin (Nissen & Sutter, 1990), blinded solutions were kept wrapped in foil throughout an experiment. To ensure even treatment during 20–30 min treatments, whole seedlings were cut from agar plates and treated by soaking their agar block in a 24‐well plate. For 100‐s timelapse movies, plants were treated on slides by being mounted in either control or IAA solution. Imaging began almost immediately; timelapse movies from both Regions 2 and 3 were collected within 7 min. For 20–30 min treatments, imaging concluded within 30 min. Because darkness can stimulate degradation of cytoskeletal organizing proteins (Dyachok *et al.*, 2011) and a reorientation of actin filaments in hypocotyls (Breuer *et al.*, 2014), plants were left under grow lights (40 μM photons m^−2^ s^−1^) while soaking during 20‐min treatments, and slides were prepared in the light. All IAA and NAA experiments were performed and analyzed double blind.

### Individual actin filament dynamics

Individual actin filaments were captured in Col‐0 root epidermal cells with 100‐s timelapse (one frame per s) VAEM using a 150 × 1.45 NA UApoN TIRF objective (Olympus). Timelapse movies and regions of interest (ROI) were analyzed in fiji is just imagej (FIJI, Madison, WI, USA, https://fiji.sc). To best display filaments and their dynamics, brightness and contrast were enhanced in the final montages of Fig. 2(b,c). Occasionally, minimal adjustments to brightness and contrast were made during analysis to more definitively follow some filaments or events. Filament severing frequency, maximum filament length, filament lifetime and elongation rates were measured as described previously (Staiger *et al.*, 2009; Henty *et al.*, 2011; Cai *et al.*, 2014; Henty‐Ridilla *et al.*, 2014). To measure bundling, unbundling and end‐to‐end annealing frequencies, we cropped *c.* 15 μm × 15 μm ROIs at random locations throughout a cell. For measuring individual filament responses to IAA, we used *c.* 7 μm × 7 μm randomly selected ROIs. To account for differences in filament density in short and long cells, bundling, unbundling and annealing frequencies were normalized against filament numbers in each ROI. For detailed methods, see Methods S4.

### Accession numbers

Sequence data relevant to this article appear in the Arabidopsis Information Resource database (https://www.arabidopsis.org/) under: AUX1 (At2G38120).

## Results

### Actin organization correlates with cell length

**Figure 1:**
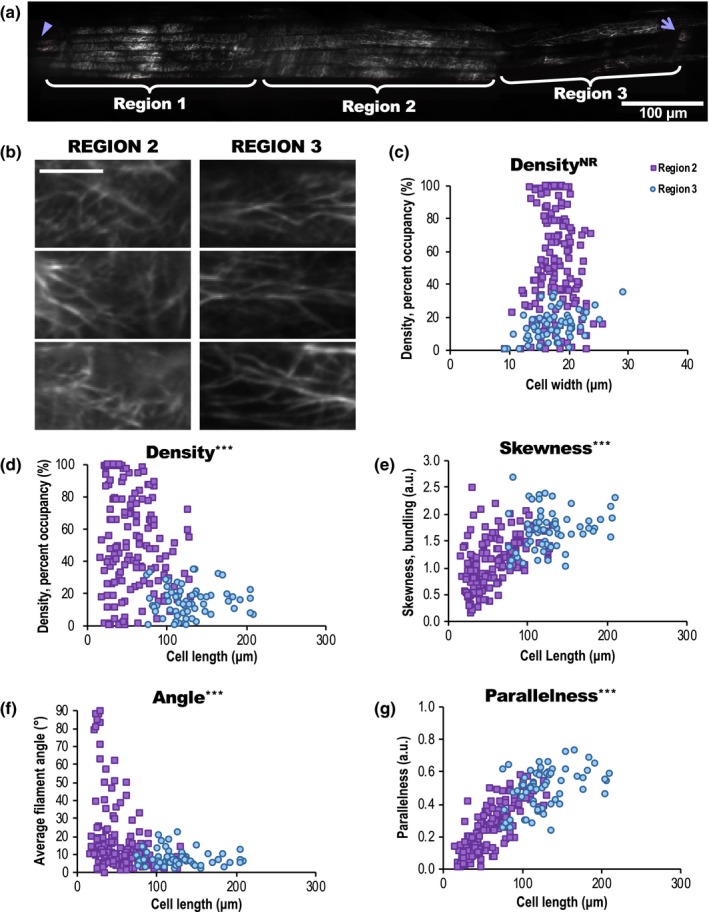
Actin organization is predictive of epidermal cell length in the root elongation zone. (a) Mosaic of root cap and elongation zone in an *Arabidopsis thaliana* seedling expressing green fluorescent protein fused to the second actin‐binding domain of Arabidopsis FIMBRIN1 (GFP‐fABD2) imaged with variable angle epifluorescence microscopy (VAEM). Arrowhead, root apex; arrow, first root hair initiation. mosaicj was used to compile 13 original VAEM images. Region 1 encompasses the root cap (root apex to *c.* 300 μm from the apex); Region 2 begins *c.* 300 μm from the apex and ends *c.* 625 μm from the apex; Region 3 begins *c.* 625 μm from the apex and ends at the first clearly visible root hair initiations, *c.* 860 μm from the apex. Bar, 100 μm. (b) Representative images of actin organization in two root regions. Bar, 10 μm. (c–g) Quantification of actin architecture or orientation in the root elongation zone, metrics plotted with respect to corresponding epidermal cell length (d–g), or cell width (c). Filament architecture and orientation were not predictable based on cell width but were highly correlated with cell length. Supporting Information Fig. S2 shows results for skewness, angle and parallelness vs cell width, which also showed no relationship, and Fig. S1 shows comparisons of mean measurements of actin from Region 2 (purple squares) and Region 3 (blue circles). Mean cell length ± 1 SD: Region 2 = 57 ± 28 μm, Region 3 = 128 ± 34 μm. *n* = 60–150 cells per region from 20 roots. a.u., arbitrary units. NR, no predictive relationship; ***, *P* ≤ 0.0001, Bivariate fit/ANOVA for all data points for each parameter. Results are from one experiment.

Actin organization in living epidermal cells of the root cap and elongation zone, examined with VAEM, displayed a consistent and previously observed pattern of organization (Baluška *et al.*, 1997; Baluška & Mancuso, 2013; Figs 1a, S1a). We wondered whether there were quantitative differences in actin organization that correlated with cell size and developmental stage. After plant cells are generated in the root meristem, they spend *c.* 4 d progressing through the meristematic region and the transition zone before entering the rapid elongation zone, where they spend only hours (Beemster & Baskin, 1998, 2000; van der Weele *et al.*, 2003). The consistent progression of aging, growing cells allows quantification and comparison of actin arrays in both the slower‐growing late meristematic/transition zone and the rapid elongation zone. Whether actin bundles inhibit (Gilliland *et al.*, 2003; Holweg *et al.*, 2004; Rahman *et al.*, 2007) or promote (Kandasamy *et al.*, 2009; Yang *et al.*, 2011; G. Li *et al.*, 2014) cell expansion remains controversial. We sought insight to the role of actin bundling in root epidermal cell expansion, because the epidermis drives tissue expansion (Savaldi‐Goldstein *et al.*, 2007 (shoots); Hacham *et al.*, 2011).

In order to quantitate whether actin array organization varies with cell size, we collected overlapping VAEM images of GFP‐fABD2‐labeled actin filaments from the root apex through the elongation zone in Col‐0 plants. The demarcation between the root cap (here called ‘Region 1’) and what appeared to be the visible transition zone (‘Region 2’) was drastic. Isotropic cells that delineate the late meristematic/early transition zone (i.e. Region 2) clearly emerge from under the rectangular cells of the root cap (i.e. Region 1). We preliminarily delineated Region 3 by an observable decrease in actin filament abundance (admittedly, a subjective criterion, but characteristics are best analyzed *en masse*). Region 1 encompassed the root apex through *c.* 300 μm from the apex; Region 2 began *c.* 300 μm from the apex through *c.* 625 μm from the apex; Region 3 began *c.* 625 μm from the apex through the first clearly visible root hair initiations, *c.* 860 μm from the apex. These regions are very close to root measurements for the root cap, transition zone and rapid elongation zone reported in Verbelen *et al.* (2006). Representative images showed conspicuous array differences between regions (Figs 1b, S1a). Aspects of actin organization, including percentage occupancy or density (100% density = an image where all pixels contain fluorescence), extent of actin filament bundling (measured as ‘skewness’ of an image's pixel intensity distribution), average filament angle relative to a cell's longitudinal axis (angle), and parallelness of filaments to each other, were quantified as described previously (Higaki *et al.*, 2010b; Ueda *et al.*, 2010; Henty *et al.*, 2011; Li *et al.*, 2012; Cai et al, 2014; Cao *et al.*, 2016). These analyses showed (Fig. S1b–e) that the cortical actin array in Region 2 was significantly denser and less bundled than in Region 3 (i.e. Region 2 exhibited a more ‘normal’ distribution of pixel intensities, whereas the distribution of pixel intensities from images of Region 3 was skewed towards more higher intensity pixels). Region 1 was similar in density to Region 2, but more bundled. Filaments and bundles in Region 3 cells were substantially more longitudinal than those in regions 1 or 2. Because root cap cells neither belong to the same epidermal cell lineage nor follow the same cell expansion gradient as regions 2 and 3, we eliminated Region 1 from further analysis.

Because of the substantial differences in actin organization among epidermal cells within the elongation zone, we hypothesized that if certain actin arrays correlate with expanding cells, cell size should predict actin organization and vice versa. Although there was no predictive relationship between cell widths and actin filament density, skewness/bundling, angle or parallelness (Figs 1c, S2), cell lengths were highly predictive of each actin metric (Figs 1d–g, S3). Short cells (Region 2) exhibited higher actin density (Fig. 1d), lower bundling (Fig. 1e) and what might be perceived as ‘disorganized’ actin, with higher average filament angles (Fig. 1f) and lower parallelness (Fig. 1g) compared with long cells (Region 3). These highly predictive relationships between cell length and each aspect of actin organization also were apparent in the WS ecotype expressing GPF‐fABD2, and in *aux1‐100* (WS background; Fig. S7, see later), whose average root epidermal cell lengths were significantly longer than WT. Although we could not consistently measure cell growth rates (based on literature such as Beemster & Baskin, 1998; van der Weele *et al.*, 2003) and capture simultaneous actin dynamics, and did not determine whether bundles promote or precede expansion, more bundling clearly occurred as cell lengths increased (Figs S3, S7, S9), demonstrating that bundles did not inhibit cell growth under control conditions.

Although adherence to a fairly linear relationship does not reveal cause‐and‐effect, actin filament parallelness was most directly correlated with cell length (*R* ^2^ = 0.68; Figs 1g, S3d). To determine whether any actin parameter explains the most variance from the mean for each cell, we performed principal component analysis (PCA) on each dataset, finding that interactions between cell length, filament parallelness and to a lesser extent, skewness, explained most of our observations for both WT ecotypes (Col‐0 and WS) and *aux1‐100* (Tables S1–S6).

Aside from correlations between actin organization and cell size, we needed to more objectively categorize WT cells into ‘Region 2’ or ‘Region 3’. By plotting each cell's specific actin metrics against its length or width, we defined maximum cell sizes for each region. We categorized cells up to 85 µm (i.e. mean cell length (57 µm) plus one SD, 28 µm) in Region 2; we categorized cells longer than 94 µm (i.e. the mean cell length (128 µm) minus 1 SD, 34 µm) as being in Region 3. Cells whose lengths fell between 85 and 94 μm were counted in both Regions 2 and 3. These cutoffs, based on our VAEM images, assigned ‘Region’ in further experiments on the Col‐0 GFP‐fABD2 lines (see the Materials and Methods section; Methods S3; Figs 3, S5–S6).

We used LSFM during an on‐campus demo to image elongation of an individual root every 15 min for 10 h (Video S1), and from that dataset, measured individual cell elongation rates from 27 cells representing both regions 2 and 3 (Fig. S11, see later). We found that cells in the root elongation zone exhibited triphasic growth. Cells < 30 μm expanded slowly, at *c.* 0.015 ± 0.004 μm min^−1^ (mean ± SE). The elongation rate of cells between 30 and 100 μm long was nearly 10× faster (*c.* 0.145 ± 0.007 μm min^−1^). Growth in cells > 100 μm appeared to slow or cease (Fig. S11, see later). Drastically different plant growth conditions (overnight inside a capillary tube for LSFM vs during the day on the agar surface for VAEM), sample size (Video S1; Fig. S11 (see later) feature a single root), and our inability to definitively discern short epidermal vs cortical cells in the LSFM movie could account for differences in region boundaries between our LSFM and VAEM data. Root hairs emerged (arrowheads, Fig. S11, see later) as cell expansion slowed, but some trichoblast elongation (cells 16, 19, and 26) continued after emergence, indicating that developmental ‘regions’ and ‘zones’ might overlap. LSFM resolution was insufficient to measure precise changes in actin organization concurrent to cell elongation, but Video S1 shows that actin arrays appear to become more bundled and longitudinal as cells elongate. Taken with our previous results, these data demonstrated that cell elongation rates accelerated along the root elongation zone where actin filament bundling and parallelness also increased (Figs 1, S3).

### Short cells are characterized by high filament end‐to‐end annealing frequencies

Cortical actin arrays constantly remodel depending on a cell's needs (Staiger *et al.*, 2009; Smertenko *et al.*, 2010; Henty *et al.*, 2011; Henty‐Ridilla *et al.*, 2013; Henty‐Ridilla *et al.*, 2014; Cao *et al.*, 2016). For example, arrays in isotropically growing cotyledon pavement cells exhibit ‘more random’ and ‘more dynamic’ arrays than the anisotropically growing cells of the root elongation zone (Smertenko *et al.*, 2010). We hypothesized that the Region 2 actin network would be more dynamic than Region 3's. We collected 100‐s timelapse movies (at one frame per s) from short and long cells in the same roots and calculated the pairwise correlation coefficient among all possible temporal intervals (Vidali *et al.*, 2010), finding that actin array dynamicity in Region 2 was significantly reduced compared to Region 3 (Fig. S4), opposite to our hypothesis. Region 2 arrays were dense, so we considered that a gross comparison of pixel intensities and occupancies among intervals of timelapse movies might not account for all dynamic behaviors.

**Figure 2:**
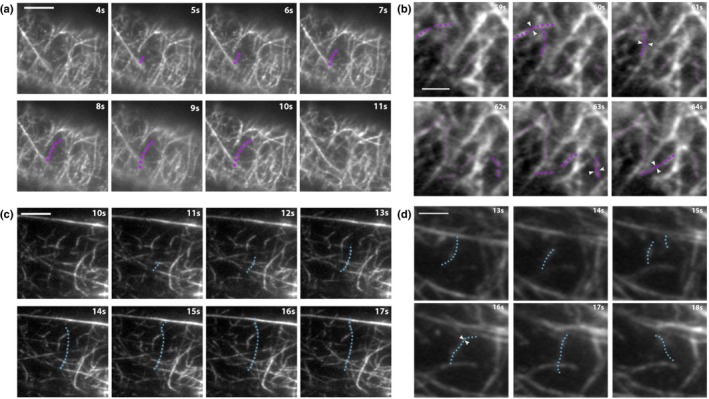
Timelapse imaging of cortical actin filaments in root epidermal cells shows differences in the dynamic behavior between short and long cells. (a, c) The cortical actin cytoskeleton in 6‐d‐old light‐grown *Arabidopsis thaliana* root epidermal cells expressing green fluorescent protein fused to the second actin‐binding domain of Arabidopsis FIMBRIN1 (GFP‐fABD2) was imaged with timelapse variable angle epifluorescence microscopy (VAEM). Representative images of individual filament dynamics in short cells (≤ 85 μm long, Region 2) and long cells (> 94 μm long, Region 3). On average, filaments in short cells (a; filament highlighted in purple) elongated over 25% more slowly and grew to be nearly 30% shorter than filaments in long cells (c; filament highlighted in blue). Severing frequencies and filament lifetimes did not vary between regions; see Table 1. Bars in (a) and (c), 5 μm. (b, d) Regions of interest (ROI; 227.7 μm^2^) were selected from the same movies as (a, c). Annealing occurs *c.* 10‐fold more frequently in short cells (b; filaments highlighted in purple) compared with long cells (d; filament highlighted in blue). Note that four annealing events (white arrowheads) occurred within 6 s in (b) compared with only one event in (d). Dots indicate fragments involved in annealing events. Quantification of annealing frequencies as well as bundling and unbundling frequencies are shown in Table 1. Although actin filament arrays in long cells were substantially more bundled compared with short cells (see Fig. 1), there were no differences in bundling or unbundling frequencies when event frequencies were calculated on a per‐minute, per‐filament basis. Bars in (b, d), 2 μm. 100‐s timelapse movies were collected from short and long cells in the same 30 roots. Note: brightness and contrast were enhanced in the montages of (b, c) to better show the filament and its changes.

In order to determine what specific properties build filament arrays in cells, we quantified individual actin filament behaviors (Li *et al.*, 2015b). We expected increased turnover in short cells and more frequent bundling in long cells. On average, filaments in short and long cells behaved in a similar way, except that long cells exhibited significantly longer, faster‐growing filaments (Fig. 2; Table 1). Actin filaments can grow by polymerization (i.e. incorporation of ATP‐loaded actin monomers) or through end‐to‐end annealing of filament segments, which is a purportedly faster, lower energy process to build filaments (Smertenko *et al.*, 2010; Li *et al.*, 2012). Upon measuring bundling, unbundling and annealing frequencies (see Methods S4), we observed no differences in bundling or unbundling frequencies, but shorter cells exhibited a multifold increase in annealing (Fig. 2; Table 1). Our results demonstrate that filament end‐to‐end annealing plays a role in the slow, isotropic expansion that occurs in short cells, but is downregulated in faster‐growing cells toward the end of the elongation zone.

**Table 1:** Individual actin filament behaviors in *Arabidopsis thaliana* root regions 2 and 3.

Parameter	Region 2	Region 3
**Maximum filament length (μm)**	**5.7 ± 0.3**	8.1 ± 0.4***
Filament lifetime (s)	23.5 ± 1.5	23.5 ± 1.2^ND^
**Elongation rate (μm s^−1^)**	**0.96 ± 0.05**	1.32 ± 0.08***
Severing frequency (breaks per μm s^−1^)	0.04 ± 0.004	0.04 ± 0.0032^ND^
Event frequency per min per filament		
Bundling^a^	0.111 ± 0.009	0.103 ± 0.0092^ND^
Unbundling	0.030 ± 0.004	0.032 ± 0.0042^ND^
**Annealing**	**0.100 ± 0.009**	0.012 ± 0.003***

Bold highlights parameters with statistically significant differences.

Values are means ± 1 SE.

Average number of actin filaments and bundles per 227.7 μm^2^ region of interest (ROI): Region 2, 98.7 ± 4.1; Region 3, 64.6 ± 2.4.

Per region, *n* ≥ 50 filaments from > 25 cells from ≥ 15 roots.

Bundling, unbundling, and annealing events: per root region, *n* = ROI (227.7 μm^2^) from a total of 30–37 cells from 30 roots. ND, no statistical differences; ***, *P* ≤ 0.001 (Student's *t*‐test).

a Bundling includes both zippering (*c.* 90% of observed bundling events) and ‘other’ (remaining *c.* 10% of observed bundling events); see the Materials and Methods section for more information.

### Actin organization responds to short‐term IAA treatments

In order to decipher which actin parameter(s) coincided with cell expansion, and find stronger indicators of causality, we treated roots with the natural auxin IAA, which inhibits root growth within minutes (Hejnowicz & Erickson, 1968; Fendrych *et al.*, 2018). Auxin affects actin organization and dynamics, and inhibits root growth (reviewed in Zhu & Geisler, 2015; Fendrych *et al.*, 2018), known to depend on an intact cytoskeleton (reviewed in Hussey *et al.*, 2006, and Li *et al.*, 2015a). Yet, actin response following short‐term auxin treatments – that is, a direct link – remains undetermined. If decreased actin density and increased bundling (i.e. ‘organized’ actin filaments) are growth hallmarks, and if auxin modulates the actin cytoskeleton, then growth inhibitory IAA should induce the opposite actin phenotype: increased density and decreased bundling. Further, if lower average filament angle and higher parallelness correlate with rapidly growing cells (described in Dyachok *et al.*, 2011), IAA should increase filament angle and decrease parallelness.

**Figure 3:**
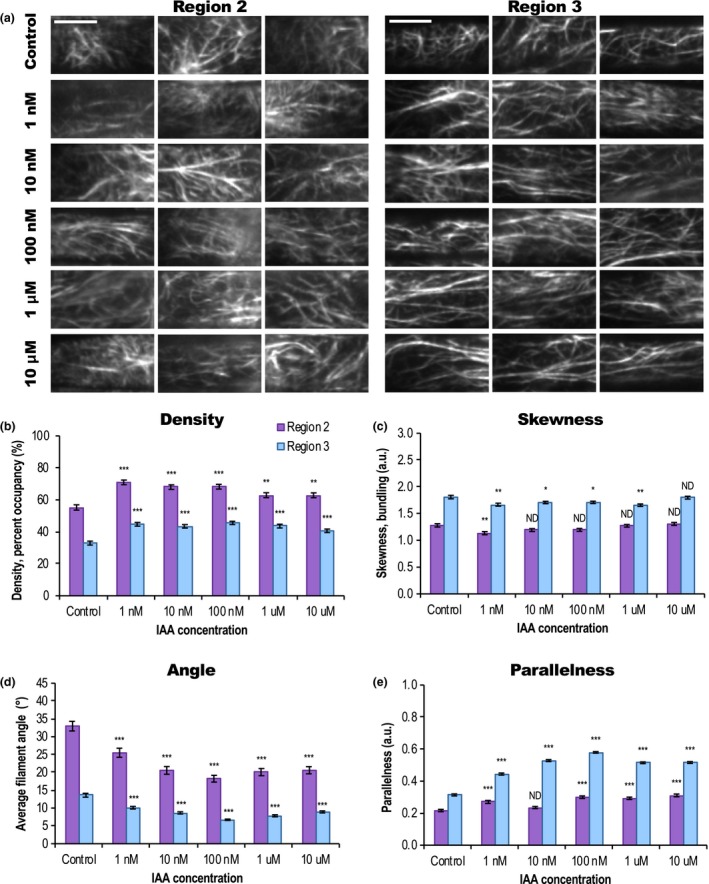
Short‐term indole‐3‐acetic acid (IAA) treatments induce changes in actin filament organization. (a) Representative variable angle epifluorescence microscopy (VAEM) images of green fluorescent protein fused to the second actin‐binding domain of Arabidopsis FIMBRIN1 (GFP‐fABD2)‐labeled actin in *Arabidopsis thaliana* epidermal cells from Region 2 (≤ 85 μm long) and Region 3 (≥ 94 μm long), treated for 20–30 min with indicated doses of IAA or control. Bars, 10 μm. (b–e) Quantification of actin architecture and orientation in root epidermal cells: IAA triggered an increase in actin filament density (b) and decrease in skewness (c). Region 2 measurements are shown in purple; Region 3 in blue. (d,e) After IAA treatments, actin arrays in both regions were more ‘organized,’ with lower average filament angle (d) relative to the longitudinal axis of the cell and filaments generally more parallel to each other (e). Changes in actin orientation (d) and (e) were dose‐dependent, see Supporting Information Fig. S5. Cells whose lengths fell between 85 and 94 μm were counted in both regions. *n* = 8–12 cells per region per root from ≥ 10 roots per treatment. Error bars represent ± 1 SE; a.u., arbitrary units. ND, no statistical differences; *, *P* ≤ 0.05; **, *P* ≤ 0.01; ***, *P* ≤ 0.0001, one‐way ANOVA, compared with Dunnett's Method, comparing doses to control in each Region, in JMP. Results are from one representative experiment of three similar experiments with similar results. All IAA experiments were performed and analyzed double blind.

As expected, 20–30 min IAA treatments significantly increased filament density and decreased bundling (Fig. 3a–c), but simultaneously induced *higher* levels of actin organization in the presumably nongrowing cells – we observed a dose‐dependent increase in longitudinal, parallel filaments (Figs 3a,d–e, S5). A timeseries experiment established that cells maintained the IAA‐induced increase in organization for ≥ 60 min from initial treatment (Fig. S6; Table S7). Increased filament density appeared before strong changes in angle and parallelness; the 30 s timeframe of IAA‐induced root growth inhibition reported in Fendrych *et al.* (2018) is much faster than gross actin reorganization was discernable, at least by current methods. Still, these are the first data that quantitatively document actin's short‐term response to moderate doses of IAA, showing that actin becomes more ‘organized’ in response to a treatment that inhibits growth.

### IAA stimulates actin filament unbundling

Links between auxin and actin clearly exist (reviewed in Zhu & Geisler, 2015) but the specific components of these pathways – and actin's role in them – are unresolved, so we evaluated actin's role in IAA response by measuring whether individual filament behaviors change in the minutes following IAA treatment.

**Figure 4:**
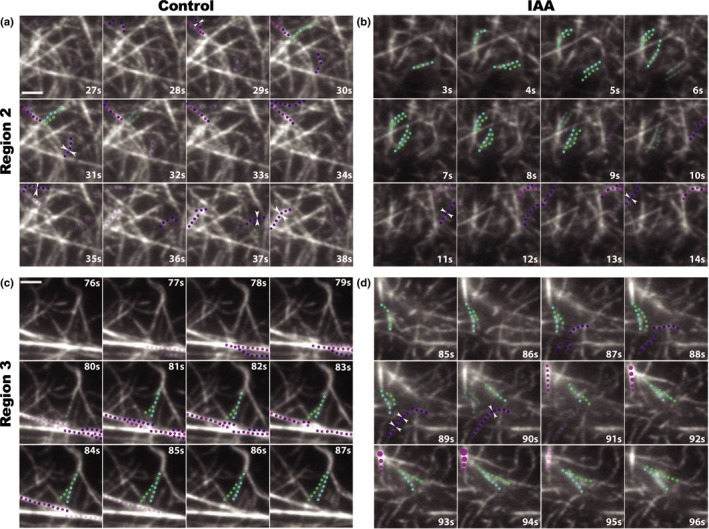
Short‐term auxin treatments cause actin filament unbundling. Representative images of individual filament bundling, unbundling, and annealing in *Arabidopsis thaliana* root Region 2 cells (a, b) and Region 3 cells (c, d); control (a, c) vs 10 nM indole‐3‐acetic acid (IAA) (b, d). Bars, 2 μm. (a, b) Timelapse series of variable angle epifluorescence microscopy (VAEM) images show that 10 nM IAA (b) increased actin filament unbundling in Region 2 within 7 min compared with control (a). Note that one unbundling event (filament unbundling shown as blue and green dots separating) occurred in (a), whereas three occurred in the same timespan in (b). There also was a small but statistically significant decrease in annealing events (white arrowheads) after IAA treatment. Other aspects of individual filament behaviors did not significantly change after treatment; for complete quantification of all measured individual filament dynamics, see Table 2. (c, d) Treatment with 10 nM IAA (d) increased actin filament unbundling and filament end annealing in Region 3 within 7 min compared with control (c). As in Region 2, IAA stimulated unbundling of actin filaments: two unbundling events are shown in (d) compared with only one event in (c). IAA also stimulated an increase in annealing in Region 3, where three annealing events are shown by white arrowheads (d). Bundling events are shown by either purple and magenta dots coming together (zippering of two independent filaments) or a series of magenta dots increasing in size (fluorescence intensity increase with no visible filament zippering). 100‐s timelapse movies were collected from short and long cells in the same 28 6‐d‐old, light‐grown roots. All auxin experiments were performed and analyzed double blind.

Because 20–30 min IAA treatments triggered increased actin density and longitudinal, parallel filaments, we hypothesized that individual filaments would quickly respond by elongating faster and increasing severing, unbundling and/or end‐to‐end annealing (a result of decreased end‐capping; Li *et al.*, 2012). We quantified actin dynamics in Col‐0 epidermal cells in regions 2 and 3 within 7 min of 10 nM IAA treatment. We observed no changes in most individual filament behaviors in either region within this 7‐min timeframe (Table 2), although the differences in filament elongation rates and maximum filament length we observed previously between regions (Table 1) were reproduced. However, we observed an IAA‐induced doubling of unbundling events in both short (Region 2) and long cells (Region 3; Fig. 4; Table 2). In long cells, IAA induced a nearly five‐fold increase in annealing (Fig. 4d; Table 2). Actin participates in short‐term responses to IAA by unbundling filaments and altering annealing frequencies within 7 min. These changes likely contribute to the gross increase in filament density observed at 20–30 min (Fig. 3).

**Table 2:** Actin filament dynamics in *Arabidopsis thaliana* roots after treatment with indole‐3‐acetic acid (IAA).

Item	Region 2		Region 3	
Parameter/treatment	Control	IAA	Control	IAA
Maximum filament length (μm)	5.2 ± 0.2	4.6 ± 0.2^ND^	9.8 ± 0.6	9.7 ± 0.4^ND^
Filament lifetime (s)	27.4 ± 1.9	24.9 ± 1.2^ND^	28.8 ± 2.2	33.9 ± 2.1^ND^
Elongation rate (μm s^−1^)	0.97 ± 0.14	0.88 ± 0.05^ND^	1.63 ± 0.06	1.62 ± 0.07^ND^
Severing frequency (breaks per μm s^−1^)	0.05 ± 0.003	0.06 ± 0.003^ND^	0.03 ± 0.002	0.03 ± 0.002^ND^
Event frequency per min per filament				
Bundling^a^	0.216 ± 0.023	0.170 ± 0.015^ND^	0.239 ± 0.023	0.187 ± 0.015^ND^
**Unbundling**	**0.092 ± 0.010**	0.218 ± 0.017***	**0.104 ± 0.012**	0.208 ± 0.025**
**Annealing**	**0.172 ± 0.009**	0.133 ± 0.013*	**0.034 ± 0.006**	0.147 ± 0.020***

Bold highlights parameters with statistically significant differences.

Values are means ± 1 SE.

Average number of actin filaments and bundles per 57.8 μm^2^ region of interest (ROI): Region 2, 48.6 ± 2.1; Region 2 + IAA, 48.4 ± 1.6; Region 3, 26.9 ± 1.1; Region 3 + IAA, 28 ± 1.3; see the Materials and Methods section.

Six‐day‐old roots were treated with 10 nM IAA or control and epidermal cells were imaged for ≤ 7 min after treatment.

Per region per treatment, *n* ≥ 50 filaments from ≥ 20 cells from ≥ 12 roots. ND, no statistical differences, Student's *t*‐test compared with control for that region.

Bundling, unbundling, and annealing events: per root region per treatment, *n* = ROI (57.8 μm^2^) from a total of 21–23 cells from 18–22 roots. *, *P* ≤ 0.05; **, *P* ≤ 0.001; ***, *P* ≤ 0.0001; ND, no statistical differences, Student's *t*‐test vs same region control‐treated.

a Bundling includes both zippering (*c.* 90% of observed bundling events) and ‘other’ (remaining *c.* 10% of observed bundling events); see the Materials and Methods section for more information. These percentages hold for both control‐ and IAA‐treated plants. All auxin experiments were performed and analyzed double blind.

### Actin arrays in *aux1* mutants are insensitive to IAA but partially respond to NAA

The auxin importer AUX1 was identified in an EMS mutant screen for resistance to auxins (Maher & Martindale, 1980; Pickett *et al.*, 1990), and the protein binds IAA with extremely high affinity (Yang *et al.*, 2006; Carrier *et al.*, 2008). AUX1 mutants are agravitropic and exhibit root elongation in the presence of IAA (which inhibits WT root growth), but the synthetic, membrane‐permeable auxin NAA inhibits *aux1* root growth to WT levels (Maher & Martindale, 1980; Pickett *et al.*, 1990; Bennett *et al.*, 1996; Marchant *et al.*, 1999; Fendrych *et al.*, 2018). Root cells in *aux1* mutants take up significantly less IAA (Rashotte *et al.*, 2003; Band *et al.*, 2014; Hayashi *et al.*, 2014; Rutschow *et al.*, 2014; Dindas *et al.*, 2018) and are larger than WT cells (Ugartechea‐Chirino *et al.*, 2010; Figs S7, S9). Intracellular auxin concentrations are higher further from the meristem, in long WT root epidermal cells (Brunoud *et al.*, 2012), possibly because IAA concentration regulates the amount of time cells spend in the elongation zone (Rahman *et al.*, 2007). We hypothesized that AUX1 might play a previously uncharacterized role in short‐term auxin signaling to the cytoskeleton.

We expressed GFP‐fABD2 in *aux1*‐*100* (WS background) and in *aux1*‐*22* (Col‐0 background; Feldmann, 1991; Roman *et al.*, 1995; Bennett *et al.*, 1996), to test the hypothesis that if AUX1 were upstream of cytoskeletal rearrangements in response to auxin, the mutants’ actin cytoskeletons would not respond to 20–30 min IAA treatments but would respond to NAA, which reaches the cytoplasm in high quantities because it is highly membrane permeable, and need not rely on an influx carrier (Delbarre *et al.*, 1996; Carrier *et al.*, 2008). Because root epidermal cells in *aux1* plants were significantly longer than WT (Figs S7, S9), analyzing actin response by ‘regions’ seemed imprecise. For example, a 120 μm‐long WT cell that will grow to a final length of 140 μm is at a different point in development than a 120 μm‐long *aux1*‐*100* cell that will ultimately grow to 290 μm. Therefore, we quantified differences in actin array on a per‐cell basis (computing one measurement per actin parameter per cell), rather than by region (see the Materials and Methods section; Methods S3; Figs S7, S9).

**Figure 5:**
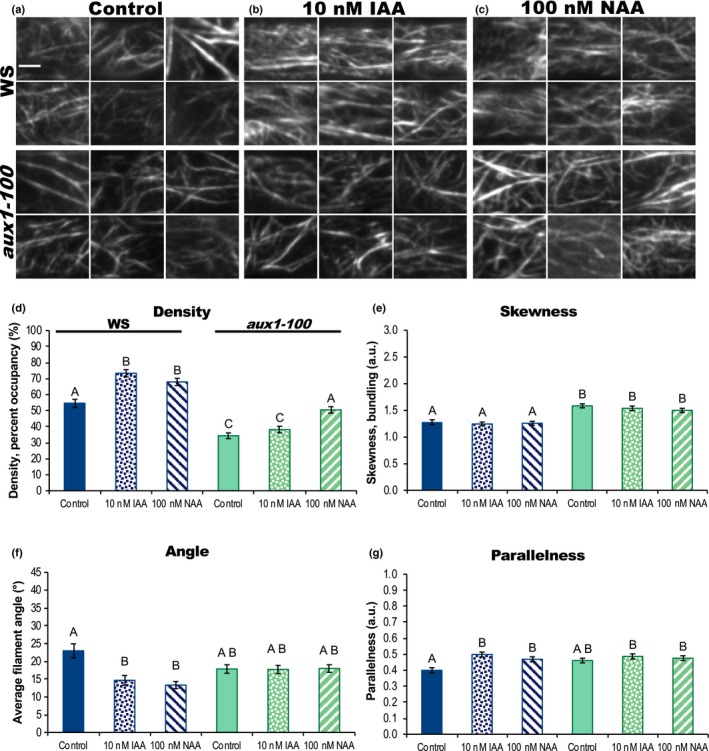
Actin organization in *aux1‐100* fails to respond to short‐term indole‐3‐acetic acid (IAA) treatments but responds partially to the membrane‐permeable auxin 1‐naphthylacetic acid (NAA). (a–c) Representative VAEM images of green fluorescent protein fused to the second actin‐binding domain of Arabidopsis FIMBRIN1 (GFP‐fABD2)–labeled actin in *Arabidopsis thaliana* epidermal cells from wild‐type (ecotype Wassilewskija, WS) and *aux1*‐*100*, treated for 20–30 min with control (a), 10 nM IAA (b), or 100 nM NAA (c). Bar, 5 μm. (d–g) Quantification of actin organization in root epidermal cells. Wild‐type response is shown in blue and aux1‐100 in green; control, solid; 10 nM IAA, dots; 100 nM NAA, stripes. IAA failed to trigger an increase in actin filament density in *aux1*‐*100* (d) but actin density in *aux1‐100* increased in response to NAA. Skewness in both genotypes did not significantly respond to either treatment (e). After IAA and NAA treatments, actin arrays in WS plants were more ‘organized,’ with lower average filament angle (f) relative to the longitudinal axis of the cell and filaments generally more parallel to each other (g). Average actin filament angle and parallelness in *aux1*‐*100* failed to reorganize in response to either IAA (dots) or the membrane‐permeable auxin NAA (stripes). *n* = 7–32 cells per root; 9–11 roots per genotype per treatment. Error bars represent ± 1 SE; a.u., arbitrary units. Different letters indicate statistically significant differences, oneway ANOVA, compared with Tukey–Kramer honest significant difference in JMP. Actin measurements were quantified on a per‐cell basis; see the Materials and Methods section for description and Supporting Information Fig. S7 for scatter plots. Results are from one representative experiment of two similar experiments with similar results. All auxin experiments were performed and analyzed double blind.

Both *aux1* mutants had mean cell lengths longer than WT, and actin organization that differed from WT under control conditions (Figs 5, S7–S9). When compared to its respective WT ecotype, each *aux1* mutant allele exhibited significantly lower average filament density and increased skewness. Filaments were more longitudinal and parallel to one another. Mutants' longer cells and ‘more organized’ actin filament organization fits the model that higher levels of apparent ‘organization’ coincide with cell expansion under control conditions.

Actin organization in WS plants expressing GFP‐fABD2 responded to short‐term IAA treatments almost identically as had Col‐0. Actin filament density and parallelness significantly increased, and average filament angle significantly decreased (Fig. 5). When quantified on a per‐cell basis, WS did not exhibit the small but statistically significant decrease in skewness (Figs 5e, S7) previously observed in Col‐0 (Fig. 3), perhaps because the ecotype itself is slightly resistant to auxin (Dharmasiri *et al.*, 2005b). The *aux1*‐*100* mutant's actin array did not significantly reorganize after IAA treatment (Fig. 5), indicating that actin cytoskeleton response to IAA required the transporter. To confirm the importance of AUX1 in IAA‐triggered actin cytoskeleton rearrangements, we tested a second allele, *aux1*‐*22*, whose actin array also failed to reorganize after IAA treatment (Fig. S8).

In order to understand whether the auxin hormone itself drives cytoskeletal reorganization, or if there is an intermediary between auxin, AUX1, and actin response, we tested the mutant's response to the highly membrane‐permeable auxin NAA. If AUX1's role is restricted to transporting IAA into the cell and auxin merely needs to enter cells to stimulate actin reorganization, NAA should suffice to induce a WT response in *aux1*‐*100* and should induce denser, more longitudinal, parallel arrays. But if NAA should fail to induce reorganization, we could deduce that the presence of auxin inside the cell is insufficient, and short‐term auxin‐to‐actin signaling requires AUX1 protein. Fig. 5 shows that WS responds in a similar way to NAA and IAA. However, compared to IAA on WT, NAA only partially restored actin organization in *aux1‐100*: the highly membrane permeable auxin stimulated only increased actin filament density (Fig. 5d) in *aux1‐100*, and did not affect filament angle or parallelness (Fig. 5f,g).

In order to confirm AUX1's importance in cytoskeletal responses to NAA, we tested NAA's effects on actin organization in *aux1‐22* and its WT, Col‐0. Recapitulating *aux1‐22*'s lack of response to IAA, actin organization in this mutant was largely impervious to NAA, and exhibited only a sizeable but statistically insignificant reduction in average filament angle and no changes in density or parallelness. Upon testing the effect of NAA on actin reorganization in Col‐0, we found that NAA stimulated increased filament density in Col‐0, but, when measured on a per‐cell basis, Col‐0 exhibited no changes in average filament angle or parallelness (Fig. S8). The Col‐0 and WS ecotypes are likely genetically divergent enough to explain why NAA provoked dissimilar actin reorganization; previous work identified ecotype‐specific transcriptional responses (e.g. for proteins involved in hormone signaling) to various environmental conditions (Schultz *et al.*, 2017). Actin organization in both *aux1‐100* and *aux1‐22* failed to respond to IAA and only partially responded to NAA. These results are the first that place AUX1 upstream of actin in short‐term auxin signaling events, as well as demonstrate that the import protein itself is required for complete actin responses to auxin.

## Discussion

We correlated actin architecture and orientation to cell lengths in expanding root epidermal cells and report the first quantitative assessment of actin responses to short‐term indole‐3‐acetic acid (IAA) treatments, establishing a baseline to enable further testing of auxin–actin signaling. We measured cell expansion rates on a growing root imaged with light sheet fluorescence microscopy (LSFM) and found that under control conditions, short epidermal cells (Region 2) grew at a rate of *c.* 0.015 μm min^−1^ and that long cells (Region 3) grew nearly 10 times as fast – at a rate of *c.* 0.145 μm min^−1^. Under control conditions and imaged with variable angle epifluorescence microscopy (VAEM), slow‐growing cells (Region 2) were characterized by dense actin arrays with high end‐to‐end annealing frequencies; fast‐growing cells (Region 3) exhibited more bundled, more parallel, more longitudinal actin arrays in which filaments elongated faster and grew longer. We found this same pattern of actin organization in the Wasilewskija (WS) ecotype and *aux1* (AUX1, AUXIN RESISTANT 1) mutants, indicating a possible causal relationship among cell length, skewness/bundling and filament parallelness.

Although auxin is a known root growth inhibitor with ties to the actin cytoskeleton (Hejnowicz & Erickson, 1968; Fendrych *et al.*, 2018; reviewed in Zhu & Geisler, 2015), its short‐term effects on actin organization were unknown previously. We documented actin responses to growth‐inhibitory IAA, and found that filaments unbundled to become more dense, longitudinal and parallel within 20–30 min, demonstrating that increased actin ‘organization’ and increased cell expansion are not directly causal. The natural auxin IAA doubled the frequency of unbundling events and stimulated region‐specific changes in end‐to‐end annealing frequencies within 7 min (Table 2). Our data appear to indicate that auxin‐induced increased filament density is a product of unbundling and end‐to‐end annealing; we did not investigate whether new actin monomers are translated within this 7 min timeframe.

We provide the first evidence that rapid actin reorganization in response to auxin requires the auxin transporter AUX1 and that cytoplasmic auxin contributes to actin reorganization. Actin arrays in *aux1* mutants are impervious to exogenous IAA – likely because auxin cannot enter cells in sufficient quantities – and only partially respond to the highly membrane‐permeable 1‐naphthylacetic acid (NAA), indicating that whereas auxin elicits some actin rearrangements, AUX1 itself is necessary for the full response. Although we do not establish a cause‐and‐effect relationship between increased actin bundling and elongating cells, our results disprove the hypothesis that actin bundles inherently inhibit cell expansion. Whether changes in actin organization precede or merely correlate with growth inhibition awaits access to new technologies that permit simultaneous visualization of actin dynamics and cell elongation at high spatiotemporal resolutions. In this regard, light sheet fluorescence microscopy seems quite promising based on preliminary results (Video S1; Fig. S11; Ovečka *et al.*, 2015).

### Actin array organization and filament behaviors correlate with cell length

It has been accepted that cell expansion requires observable actin ‘organization’ (Smertenko *et al.*, 2010; Dyachok *et al.*, 2011). However, ‘organization’ can take many forms and what form drives growth is unknown. Indeed, we found in two ecotypes – and in the significantly longer cells of *aux1‐100* – that filament parallelness, cell length and skewness affect one another to similar extents, producing predictable actin organization across the root elongation zone. Under control conditions, actin bundling increases as cell length increases (Figs 1, S11), and longer cells exhibit faster elongation rates along the elongation zone (Video S1; Fig. S11). Together, these data demonstrate that filament bundles do not inhibit expansion.

Although aspects of individual filament dynamics have been measured in Arabidopsis root epidermal cells (Smertenko *et al.*, 2010), we quantified additional behaviors, aiming to discern what behaviors might contribute to axial cell expansion. Actin arrays in Region 2 were significantly denser than in Region 3, so we expected a higher rate of filament turnover: increased severing, shorter filaments and filament lifetimes, and faster elongation rates. We had previously seen (Figs 1, S1) that filament arrays in Region 3 were more bundled than in Region 2 (Figs 1, S1), so we expected higher bundling or lower unbundling frequencies in longer cells, or higher unbundling frequencies in shorter cells. Both short and long cells exhibited similar individual filament behaviors (Table 1), the only major differences being shorter cells’ reduced maximum filament lengths and elongation rates, and multifold‐increased end‐to‐end annealing frequencies. In etiolated hypocotyls, increased filament lengths and lifetimes correlate with longer cells and occur in cells at the hypocotyl base, which have ceased expanding (Henty‐Ridilla *et al.*, 2013; J. Li *et al.*, 2014). Although shorter root epidermal cells have shorter average filament lengths, filament lifetimes are statistically equivalent in both regions (Table 1), and longer root cells are actually growing faster (Fig. S11; Video S1); therefore, a connection between longer lifetime and increased cell length is not evident in roots.

The most marked filament behavior difference between short and long cells was the ≤ 10‐fold higher end‐to‐end annealing frequency (i.e. number of incidents min^−1^ per filament) observed in shorter cells. Short cells exhibited higher annealing frequencies than long cells (and auxin increased annealing frequencies in long cells), suggesting that annealing serves a purpose related to growth control and/or signaling. Annealing *in vitro* is generally a function of actin concentration, filament length and/or filament end availability (Andrianantoandro *et al.*, 2001), and *in vivo* is downregulated by capping protein (CP; Li *et al.*, 2012; Henty‐Ridilla *et al.*, 2014; Li *et al.*, 2015b). Annealing builds filaments quickly and without intensive energy inputs (Smertenko *et al.*, 2010; Li *et al.*, 2012). Because maximum filament length is reduced in short cells, which also exhibit higher annealing frequencies under control conditions, and because these events seem transient, mostly annealing for only a few frames, they do not appear to build longer filaments. Perhaps shorter cells, located in the transition zone subsection of the elongation zone, undergo more cytoplasmic changes (e.g. in trafficking or vesicle distribution) in preparation for rapid elongation. Cytoplasmic changes, potentially including redistribution of auxin transporters, could also explain why auxin induces increased annealing in long cells. Our data support the possibility that an increase in end capping by CP (i.e. a decrease in the frequency of end‐to‐end annealing events) correlates with rapid growth (Region 3), whereas a downregulation of CP (and accordant increases in end‐to‐end annealing) correlates with slow growth (Region 2 exhibited high annealing frequencies under control conditions) or growth cessation (IAA induced a significant increase in the frequency of end‐to‐end annealing events).

### Actin organization predicts cell length under control conditions but not otherwise

The effect of actin bundles on growth as well as auxin's short‐term effects on actin organization were unknown previously. Studies report that long‐term, high auxin doses bundle actin filaments (G. Li *et al.*, 2014; Scheuring *et al.*, 2016), whereas short‐term, high doses stimulate growth and induce filament unbundling of mTalin–bundled actin filaments in dark‐grown rice coleoptiles (Holweg *et al.*, 2004; Nick *et al.*, 2009). Auxin transport inhibitors (e.g. 2,3,5‐triiodobenzoic acid) have the opposite effect, inducing bundling within minutes (Dhonukshe *et al.*, 2008) and inhibiting cell elongation (Rahman *et al.*, 2007). Here, we demonstrated that short‐term, growth‐inhibitory IAA treatments at doses closer to endogenous levels (Hejnowicz & Erickson, 1968; Band *et al.*, 2012; Fendrych *et al.*, 2018), decreased extent of overall filament bundling and induced increased density, longitudinality, and parallelness within 20–30 min. That the cytoskeleton looks ‘organized’ – more longitudinal and parallel – in response to IAA, which inhibits growth within minutes (Hejnowicz & Erickson, 1968; Fendrych *et al.*, 2018), definitively demonstrates that increased ‘organization’ does not inherently contribute to expansion. By showing that as cell lengths and elongation rates increase, so too does extent of actin bundling (Figs 1, S1, S3), we have disproven the hypothesis that bundles inhibit expansion (Gilliland *et al.*, 2003; Holweg *et al.*, 2004; Rahman *et al.*, 2007). The converse of the idea that bundles inhibit growth is that an absence of bundles coincides with growth. This notion is posited in studies that show that auxin inhibitors bundle filaments and inhibit growth (e.g. Dhonukshe *et al.*, 2008), and that growing cells in the hypocotyl apex have denser actin arrays than the nongrowing cells in the hypocotyl base, whose actin is more bundled (e.g. Henty *et al.*, 2011). Auxin inhibits root growth; by finding that auxin rapidly induces actin unbundling (Table 2) and overall denser filament arrays (Figs 3, 5, S5–S9), we demonstrate that an absence of bundles does not coincide with growth. Our correlative study has determined that there is no absolute relationship between actin bundling and cell expansion. Establishing cause‐and‐effect will require simultaneously capturing actin dynamics and cell elongation.

### AUX1 is necessary for actin response to auxin

Multiple auxin–actin pathways (reviewed in Overvoorde *et al.*, 2010, and Grones & Friml, 2015) assign various roles to actin in auxin response (e.g. repositioning auxin transport proteins, or inhibiting endocytosis of auxin transporters), and the short‐term pathway linking auxin and actin was thought to be the extracellular auxin receptor AUXIN BINDING PROTEIN 1 (ABP1) (Xu *et al.*, 2010, 2011; Nagawa *et al.*, 2012). The ABP1 model posits that the extracellular protein, with its coreceptor TRANSMEMBRANE KINASE, binds auxin molecules and transduces the extracellular auxin signal to Rho‐like GTPase of plants (ROPs) (Lin *et al.*, 2012; Nagawa *et al.*, 2012), inducing dose‐dependent ROP activation (Xu *et al.*, 2014). Activated ROPs then regulate actin polymerization towards growth‐related effects by inhibiting PIN endocytosis to maintain auxin export (Sauer & Kleine‐Vehn, 2011; Lin *et al.*, 2012; Nagawa *et al.*, 2012; Xu *et al.*, 2014; Zhu & Geisler, 2015). Now that ABP1's role in auxin–actin signaling is in doubt (Dai *et al.*, 2015; Gao *et al.*, 2015), the mechanism of how actin rapidly perceives auxin requires reanalysis.

Before this work, AUX1 was not suspected of being a ‘transceptor’ upstream of actin organization. AUX1 transports auxin (Bennett *et al.*, 1996; Dindas *et al.*, 2018), has homologs in all plants (reviewed in Swarup & Péret, 2012), is necessary for root gravitropism (Maher & Martindale, 1980; Marchant *et al.*, 1999; Swarup *et al.*, 2004), and is responsible for 80% of IAA uptake in root hairs (Dindas *et al.*, 2018) and rapid growth inhibition by IAA (Fendrych *et al.*, 2018). Auxin‐induced transcriptional regulation requires the SCF^TIR1/AFB^ complex, which also participates in short‐term intracellular responses to auxin (Dharmasiri *et al.*, 2005a,b; Calderón Villalobos *et al.*, 2012). Within 10 min of an auxin signal, an H^+^ influx depolarizes the plasma membrane (PM) and reduces cytosolic pH; shortly thereafter, increased intracellular Ca^2+^ propagates through the root (Dindas *et al.*, 2018).

Differences in response to IAA vs NAA, observed in both wild‐type (WT) and *aux1* roots, emphasize a role for cytoplasmic auxin in cytoskeletal reorganization. Both IAA and NAA are substrates of AUX1 (Yang *et al.*, 2006; Carrier *et al.*, 2008) and SCF^TIR1/AFB^, and protein affinity is higher for IAA than NAA (Dharmasiri *et al.*, 2005b; Calderón Villalobos *et al.*, 2012; Dindas *et al.*, 2018). Both auxins increase actin filament density in WT cells, likely through filament unbundling and, perhaps, end‐to‐end annealing. NAA stimulated disparate, ecotype‐dependent effects on actin reorganization: whereas WS responded in a similar way to NAA and IAA, Col‐0 exhibited no changes in extent of longitudinal, parallel filaments at 20–30 min. Col‐0 possibly responds to NAA more slowly than to IAA. Natural and synthetic auxins inhibit root elongation to different extents, depending on ecotype (Delker *et al.*, 2010). Differential responses to NAA have been detected across ecotypes; for example, NAA stimulates more lateral roots in 9‐d‐old Col‐0 plants vs WS or Landsberg erecta (Falasca & Altamura, 2003). Additionally, IAA and NAA can induce in Col‐0 different levels of downstream gene expression (Yoshimitsu *et al.*, 2011). Ecotype differences in signaling and physiological responses may be more prevalent than previously appreciated.

**Figure 6:**
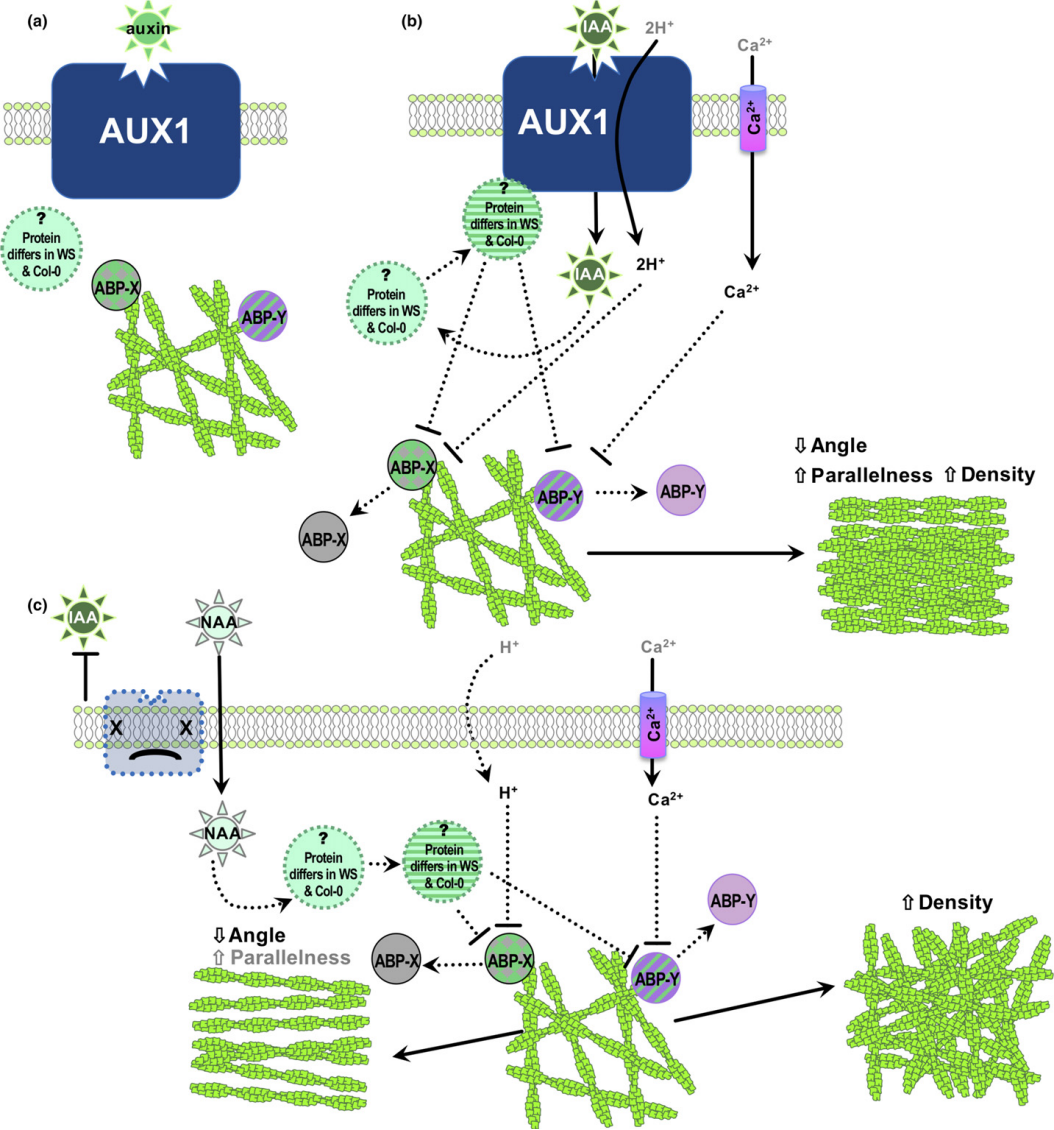
Hypothetical model of auxin perception by AUXIN RESISTANT 1 (AUX1) upstream of actin cytoskeleton reorganization. (a) Control conditions: unidentified actin binding proteins (ABP) ‐X and ‐Y are active and maintain actin array; an unidentified intermediary that differs between Col‐0 and WS is inactive. (b) Auxin is transported into a cell by AUX1, activating the unknown intermediary, inactivating both ABP‐X and ABP‐Y, and inducing increased actin filament abundance, decreased filament angle, and increased parallelness. (c) In the absence of AUX1, large quantities of indole‐3‐acetic acid (IAA) cannot enter cells. The membrane permeable auxin 1‐naphthylacetic acid (NAA) enters a cell, possibly activating the unknown intermediary; ABP‐X and ABP‐Y are differentially regulated and only either an increase in actin abundance or decrease in filament angle occurs.

Actin reorganization in both alleles of *aux1* was resistant to IAA and exhibited only partial responses to NAA, implicating AUX1 and cytoplasmic auxin as major players in auxin signaling to actin. After NAA treatments, density increased in *aux1‐100* and angle appeared somewhat (*c.* 14%), although not statistically significantly, decreased in *aux1‐22*. Attenuated actin reorganization of *aux1* in response to NAA supports the Dindas *et al.* (2018) model of an AUX1‐reliant intracellular feedback loop. IAA and NAA typically alter intracellular pH and Ca^2+^ concentrations via AUX1; these responses are reduced or absent in *aux1* mutants (Dindas *et al.*, 2018). Both H^+^ and Ca^2+^ can regulate actin binding proteins. Perhaps IAA transport through AUX1 regulates intracellular players which drive, separately, each aspect of actin reorganization (density, angle, parallelness; Fig. 6, discussed below). Alternatively, actin reorganization might be delayed in the mutants. Regardless, Dindas *et al.* (2018) and our data showing *aux1* mutants' partial actin responses to NAA demonstrate that cytoplasmic auxin itself acts as an intracellular signaling molecule that stimulates rapid cellular responses including substantial actin reorganization, deviating from the ABP1 model that relied on auxin perception at the PM and subsequent signal amplification.

### Potential players in the actin–auxin connection

Actin bundling proteins, through their ability to alter activity in response to intracellular second messengers, are likely transducers of auxin signals into cytoskeletal changes. Long‐term auxin responses in rice require the actin binding protein RMD (Rice Morphology Determinant; FORMIN5, Arabidopsis homolog FORMIN14), which is downstream of AUXIN RESPONSE FACTORS (Zhang *et al.*, 2011; G. Li *et al.*, 2014). Less is known about auxin's effect on actin during cellular activities that occur within minutes, such as polarized growth or gravitropism (Xu *et al.*, 2014; Zhu *et al.*, 2016). Auxin stimulates short‐term intracellular responses (e.g. H^+^ and Ca^2+^ influx) that occur independent of transcriptional responses and are AUX1‐dependent (Monshausen *et al.*, 2011; Dindas *et al.*, 2018). Specific actin binding proteins whose activity is modulated by these second messengers, such as the severing/bundling factors ADF or villin (Zou *et al.*, 2019), could be targets of auxin signaling. Guided by specific features of actin response to auxin (e.g. increased unbundling and end‐to‐end annealing), actin binding protein mutants could be evaluated for altered growth or actin responses in response to IAA. Auxin‐induced actin reorganization could require more than one actin binding protein; ecotype‐specific differences might explain Col‐0 and WS's dissimilar responses to NAA (Fig. 6 proposes a tentative model). The model proposes that second messengers taken up with (H^+^) or shortly after (Ca^2+^) IAA modulate activity of two actin binding proteins, leading to filament unbundling and changes in annealing frequency that cause overall increases in filament density, longitudinality and parallelness (Fig. 6b). An unidentified protein that differs in Col‐0 and WS – or slight differences in the target actin binding protein itself – may cause the ecotype‐specific responses to NAA that occur in the absence of AUX1. In the absence of AUX1, the unidentified protein and/or differences in H^+^ import, differentially regulate downstream actin binding protein(s). Due to its role in regulating end‐to‐end annealing, we suspect that CP is one of the unidentified actin binding proteins acting in our hypothetical model (Fig. 6). Under control conditions, annealing frequency is high in Region 2 and low in Region 3, implying a growth‐related gradient of CP or CP regulation. IAA stimulates increased annealing in Region 3 but less significant changes in annealing in Region 2.

Extracellular pH affects auxin uptake, where cells take up less IAA or NAA as external solution pH increases (Raven, 1975; Shinohara *et al.*, 2006; Yang *et al.*, 2006; Dindas *et al.,* 2018). By applying 20–30 min auxin treatments in high pH (pH > 10) ½ Murashige & Skoog solution, we found that WS actin reorganization in responses to both IAA and NAA were substantially reduced compared to actin responses in our other experiments (cf Fig. S10 with WS responses to IAA and NAA at *c.* pH 5.8, shown in blue in Fig. 5), although we did not test the effect of different pH solutions head‐to‐head. This result implies that cytoplasmic auxin is a key instigator of actin reorganization, which could be due to direct or indirect action on actin, particular actin binding proteins, and/or their effectors.

### The actin–auxin connection and cell expansion

The mechanisms by which auxin and actin control growth remain enigmatic. Actin could provide tracks for trafficking or prevent endocytosis of auxin transporters, alter vacuole morphology, and/or operate through another mechanism. Root cells newly differentiated from the meristem (where intracellular auxin levels are low; Brunoud *et al.*, 2012) expand isotropically. Using VAEM, we were unable to visualize actin in these youngest cells because they are covered by the root cap (Region 1). We found that actin in WT Region 2 cells – the visible epidermal cells having most recently undergone isoptropic expansion – was very dense and haphazard with few bundles, and that as cell lengths increased, their actin arrays became sparser and more bundled, longitudinal, and parallel (Figs 1, S1–S3). Very likely increased actin bundling and parallelness occur coincident with rapid axial cell elongation (Video S1; Fig. S11). Auxin regulates how long a cell spends in the elongation zone (Rahman *et al.*, 2007), and therefore how long it elongates. An initial, low, endogenous auxin signal could force young cells to break symmetry, with actin filaments becoming more longitudinal and parallel to direct vesicles to end walls for incorporation into anticlinal walls causing, ultimately, cell elongation. Whether diffusely growing cells elongate by depositing material along the entire length of the cell or primarily towards anticlinal end walls is currently unknown but could explain the purpose of longitudinal bundles.

Turgor pressure exerted by the vacuole is a primary driver of cell expansion (Cosgrove, 2005; Kroeger *et al.*, 2011; Braidwood *et al.*, 2014; Guerriero *et al.*, 2014). Six‐hour NAA treatments cause actin‐dependent vacuole constriction that ultimately reduces cell lengths (Scheuring *et al.*, 2016). If the same mechanism impels rapid growth cessation, a denser, more longitudinal actin array might effect vacuole constriction. Alternatively, cytoplasmic streaming and vesicle delivery could require an equilibrium of available tracks and space, and any actin array that disrupts that balance (i.e. unbundling to build a denser array) quickly alters cell expansion. Toward this idea, Tominaga *et al.* (2013) showed that chimeric myosins that drive faster intracellular activity grow larger plants with longer cells. We found that exogenous auxin treatments of WT plants rapidly increased actin filament density via filament unbundling. Substantial increases in filament density occur in response to other exogenous stimuli, which are also correlated with growth cessation, such as microbe‐associated molecular pattern (MAMPs) These results indicate that, like MAMP perception (Cárdenas *et al.*, 1998; Henty‐Ridilla *et al.*, 2014; Li *et al.*, 2015b), actin filaments are first responders to extracellular signals. Coping with exogenous stimuli requires cells to undertake new tasks (e.g. receptor internalization, transporter trafficking, or callose deposition) and could require increased actin density to meet them.

The hormone AUX1 and auxin efflux carriers (PINs) require actin for targeted subcellular localization (Kleine‐Vehn *et al.*, 2006, 2008). However, AUX1 is not redistributed in response to NAA (Kleine‐Vehn *et al.*, 2006), which stimulates at least partial actin reorganization in Col‐0, WS and both *aux1* mutants. Use of a photoconvertible PIN2 shows that it is not maintained at root epidermal cell PMs after auxin treatments, and most PIN2 in brefeldin A compartments is newly synthesized, not recycled (Jásik *et al.*, 2016). This complicates actin's role in auxin response. Initial auxin‐induced actin reorganization could primarily transduce an auxin signal (i.e. provide tracks to transport signaling elements into the nucleus), and be incidental to altered growth rates rather than drive growth *per se*. Wild‐type roots cease elongating within 30 s of IAA, IAA does not significantly affect *aux1‐100* elongation, but NAA reduces mutant growth rates to WT levels (Fendrych *et al.*, 2018). We found NAA induced in each *aux1* allele partial, divergent, actin reorganization. If overall actin organization and cell expansion were directly causative, NAA should have induced in *aux1* the complete reorganization observed in WT cells, or at least similar responses in both mutant alleles.

Our IAA treatments evidence that the actin cytoskeleton in root elongation zone cells responds to the growth cessation signal within minutes by significantly *increasing* filament abundance plus longitudinal, parallel filaments, opposing the view that organization coincides with expansion. We show that IAA‐induced actin rearrangements require AUX1, whereas our NAA results show that auxin itself acts as a cytoplasmic signal to modulate actin cytoskeleton organization. However auxin is acting, the relationship between actin organization and cell expansion cannot be explained by a simple model of ‘organized’ or ‘disorganized’ actin, or by the presence/absence of longitudinal bundles.

## Author contributions

RSA and CJS conceived the project and designed the experiments; RSA performed the experiments and data analysis; and RSA and CJS wrote the article.

## Supporting information

Please note: Wiley‐Blackwell are not responsible for the content or functionality of any Supporting Information supplied by the authors. Any queries (other than missing material) should be directed to the *New Phytologist* Central Office.

**Fig. S1** *Arabidopsis thaliana* epidermal cells in different root regions exhibit distinct actin filament arrays.
**Fig. S2** Actin filament arrays are not predictive of cell width.
**Fig. S3** Actin filament arrays are predictive of cell length.
**Fig. S4** Actin arrays in *A. thaliana* root Region 3 are more dynamic than in Region 2.
**Fig. S5** Short‐term IAA treatments induce dose‐dependent changes in actin filament organization.
**Fig. S6** Short‐term IAA treatments induce a time‐dependent increase in actin filament density and longitudinal orientation.
**Fig. S7** Actin filament organization plotted with respect to corresponding cell length in WS and *aux1‐100*.
**Fig. S8** Actin organization in *aux1‐22* fails to respond to short‐term IAA treatments but partially responds to the membrane‐permeable auxin NAA.
**Fig. S9** Actin filament organization plotted with respect to corresponding cell length in Col‐0 and *aux1‐22*.
**Fig. S10** Actin organization in WT WS fails to respond to short‐term auxin treatments at high pH.
**Fig. S11** Epidermal cell elongation rate increases as cells undergo axial expansion in the *A. thaliana* root elongation zone.
**Methods S1** LSFM imaging and analysis.
**Methods S2** Genotyping primers.
**Methods S3** Detailed methods for quantitative analysis of cortical actin array organization.
**Methods S4** Detailed methods for individual actin filament dynamics.
**Table S1** Eigenvectors for principal component analysis of cell size vs actin parameters in *A. thaliana* Col‐0.
**Table S2** Eigenvalues for principal component analysis of cell size vs actin parameters in *A. thaliana* Col‐0.
**Table S3** Eigenvectors for principal component analysis of cell size vs actin parameters in *A. thaliana* WS.
**Table S4** Eigenvalues for principal component analysis of cell size vs actin parameters in *A. thaliana* WS.
**Table S5** Eigenvectors for principal component analysis of cell size vs actin parameters in *A. thaliana aux1*‐*100*.
**Table S6** Eigenvalues for principal component analysis of cell size vs actin parameters in *A. thaliana aux1*‐*100*.
**Table S7** Actin organization measurements in *A. thaliana* roots after IAA treatments.

**Video S1** Maximum projection of root epidermal cell elongation within the elongation zone over 10 h.
