# Supplementary material for: Auxin‐induced actin cytoskeleton rearrangements require AUX1
Source: New Phytol. 2020 Feb 11;226(2):441–59. doi: 10.1111/nph.16382 (PMC7154765; doi:10.1111/nph.16382)
Supplement: Supplementary file 1 — Fig. S1 Arabidopsis thaliana epidermal cells in different root regions exhibit distinct actin filament arrays. Fig. S2 Actin filament arrays are not predictive of cell width. Fig. S3 Actin filament arrays are predictive of cell length. Fig. S4 Actin arrays in A. thaliana root Region 3 are more dynamic than in Region 2. Fig. S5 Short‐term IAA treatments induce dose‐dependent changes in actin filament organization. Fig. S6 Short‐term IAA treatments induce a time‐dependent increase in actin filament density and longitudinal orientation. Fig. S7 Actin filament organization plotted with respect to corresponding cell length in WS and aux1‐100. Fig. S8 Actin organization in aux1‐22 fails to respond to short‐term IAA treatments but partially responds to the membrane‐permeable auxin NAA. Fig. S9 Actin filament organization plotted with respect to corresponding cell length in Col‐0 and aux1‐22. Fig. S10 Actin organization in WT WS fails to respond to short‐term auxin treatments at high pH. Fig. S11 Epidermal cell elongation rate increases as cells undergo axial expansion in the A. thaliana root elongation zone. Methods S1 LSFM imaging and analysis. Methods S2 Genotyping primers. Methods S3 Detailed methods for quantitative analysis of cortical actin array organization. Methods S4 Detailed methods for individual actin filament dynamics. Table S1 Eigenvectors for principal component analysis of cell size vs actin parameters in A. thaliana Col‐0. Table S2 Eigenvalues for principal component analysis of cell size vs actin parameters in A. thaliana Col‐0. Table S3 Eigenvectors for principal component analysis of cell size vs actin parameters in A. thaliana WS. Table S4 Eigenvalues for principal component analysis of cell size vs actin parameters in A. thaliana WS. Table S5 Eigenvectors for principal component analysis of cell size vs actin parameters in A. thaliana aux1‐100. Table S6 Eigenvalues for principal component analysis of cell size vs actin parameters in A. thaliana aux1‐ [file NPH-226-441-s001.pdf]

New Phytologist

New Phytologist Supporting Information

Article title: Auxin-induced actin cytoskeleton rearrangements require AUX1
Authors: Ruthie S. Arieti and Christopher J. Staiger
Article acceptance date: 10 December 2019

The following Supporting Information is available for this article:

Fig. S1 *Arabidopsis thaliana* epidermal cells in different root regions exhibit distinct actin filament arrays.

Fig. S2 Actin filament arrays are not predictive of cell width.

Fig. S3 Actin filament arrays are predictive of cell length.

Fig. S4 Actin arrays in *Arabidopsis thaliana* root Region 3 are more dynamic than in Region 2.

Fig. S5 Short-term IAA treatments induce dose-dependent changes in actin filament organization.

Fig. S6 Short-term IAA treatments induce a time-dependent increase in actin filament density and longitudinal orientation.

Fig. S7 Actin filament organization plotted with respect to corresponding cell length in WS and *aux1-100*.

Fig. S8 Actin organization in *aux1-22* fails to respond to short-term IAA treatments but partially responds to the membrane-permeable auxin NAA.

Fig. S9 Actin filament organization plotted with respect to corresponding cell length in Col-0 and *aux1-22*.

Fig. S10 Actin organization in wildtype WS fails to respond to short-term auxin treatments at high pH.

Fig. S11 Epidermal cell elongation rate increases as cells undergo axial expansion in the *Arabidopsis thaliana* root elongation zone.

Table S1 Eigenvectors for principal component analysis of cell size vs. actin parameters in *Arabidopsis thaliana* ecotype Col-0.

Table S2 Eigenvalues for principal component analysis of cell size vs. actin parameters in *Arabidopsis thaliana* ecotype Col-0.

Table S3 Eigenvectors for principal component analysis of cell size vs. actin parameters in *Arabidopsis thaliana* ecotype WS.

Table S4 Eigenvalues for principal component analysis of cell size vs. actin parameters in *Arabidopsis thaliana* ecotype WS.

Table S5 Eigenvectors for principal component analysis of cell size vs. actin parameters in *Arabidopsis thaliana aux1-100.*

Table S6 Eigenvalues for principal component analysis of cell size vs. actin parameters in *Arabidopsis thaliana aux1-100.*

Table S7 Actin organization measurements in *Arabidopsis thaliana* roots after IAA treatments.

Methods S1 Detailed Methods for Long-term Light Sheet Fluorescence Microscopy.

Methods S2 Genotyping Primers.

Methods S3 Detailed Methods for Quantitative Analysis of Cortical Actin Array Organization.

Methods S4 Detailed Methods for Individual Actin Filament Dynamics.

Video S1 Maximum projection of root epidermal cell elongation within the elongation zone over 10 h.

Supporting Information References

Fig. S1 *Arabidopsis thaliana* epidermal cells in different root regions exhibit distinct actin filament arrays. **(a)** Representative VAEM images of GFP-fABD2–labeled actin in epidermal cells from subjective root regions. Region 1 encompasses the root cap (root apex through *c.* 300 µm from the apex); Region 2 begins *c.* 300 µm from the apex through *c.* 625 µm from the apex; Region 3 begins *c.* 625 µm from the apex through the first clearly visible root hair initiations, *c.* 860 µm from the apex. Scale bar, 10 µm. **(b)** to **(e)** Quantification of individual actin architecture or orientation metrics in three root regions. Results for density **(b)**, skewness **(c)**, angle **(d)**, and parallelness **(e)** showed significantly different actin arrays for most parameters, most notably between Regions 2 and 3. Actin filaments in Region 1 were dense and moderately bundled, with high average filament angle and low parallelness. Region 2 was characterized by a dense filament array, lower bundling, high average filament angle and filaments that were moderately parallel to each other. Region 3 was half as dense as Region 1 or 2, and exhibited a high degree of parallel, longitudinal bundles, with *c.* 50% decrease in average filament angle and 40% increase in filament parallelness compared with Region 2. N = 8–12 cells per region per root for 20 roots. Error bars represent ± one standard error; a.u., arbitrary units. ***, $p \leq 0.001$; ND, no statistical differences; Student’s t-test.

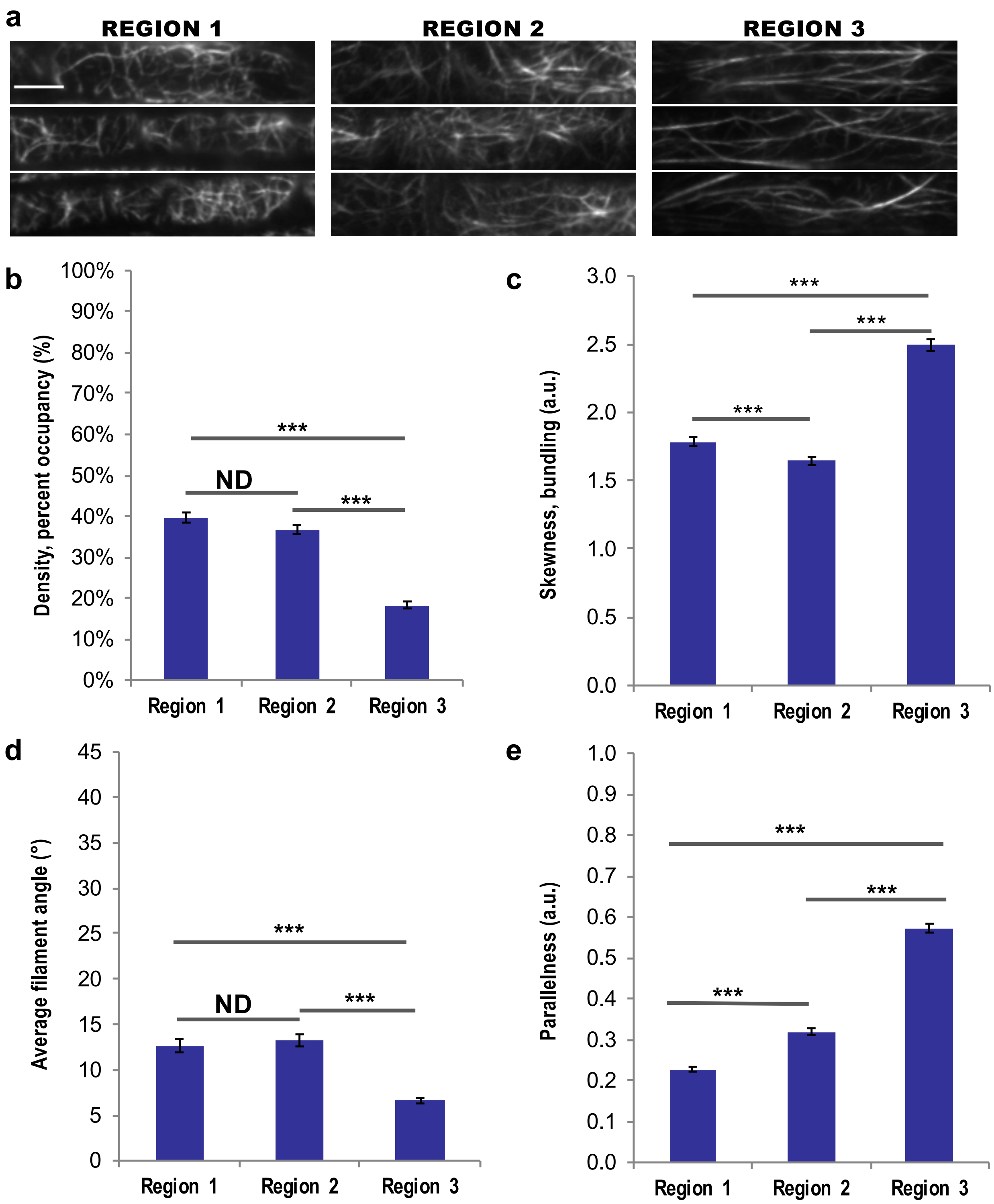
a
REGION 1
REGION 2
REGION 3
b
Density, percent occupancy (%)
100%
90%
80%
70%
60%
50%
40%
30%
20%
10%
0%
***
ND
***
Region 1
Region 2
Region 3
c
Skewness, bundling (a.u.)
3.0
2.5
2.0
1.5
1.0
0.5
0.0
***
***
***
Region 1
Region 2
Region 3
d
Average filament angle (°)
45
40
35
30
25
20
15
10
5
0
***
ND
***
Region 1
Region 2
Region 3
e
Parallelness (a.u.)
1.0
0.9
0.8
0.7
0.6
0.5
0.4
0.3
0.2
0.1
0.0
***
***
***
Region 1
Region 2
Region 3

Fig. S2 Actin filament arrays are not predictive of cell width. **(a)** to **(c)** Quantification of individual actin organization metrics plotted with respect to corresponding cell width in two *Arabidopsis thaliana* root regions. Despite the high predictability between actin architecture parameters and cell length (shown in **Fig. 1**) Filament architecture and orientation were not predictable based on cell width. Results for density **(Fig. 1)** skewness **(a)**, angle **(b)**, and parallelness **(c)** vs. cell width showed no predictive relationships. Mean cell length ± one standard deviation, Region 2 = 57 ± 28 µm. Mean cell length, Region 3 = 128 ± 34 µm. Region 2 measurements are shown in purple squares; Region 3 in blue circles. *Not shown,* ± one standard error: mean actin filament density: Region 2 = 52.3 ± 0.02%; Region 3 = 15.4 ± 0.01%. Mean actin filament bundling/skewness: Region 2 = 1.12 ± 0.03; Region 3 = 1.71 ± 0.04. Mean filament angle: Region 2 = 17.5 ± 1.6°; Region 3 = 8.1 ± 0.5°. Mean filament parallelness: Region 2 = 0.24 ± 0.01; Region 3 = 0.50 ± 0.02. N = 60–150 cells from 20 roots. A.u., arbitrary units. NR, no predictive relationship, Bivariate fit/ANOVA. Results are from one experiment.

a

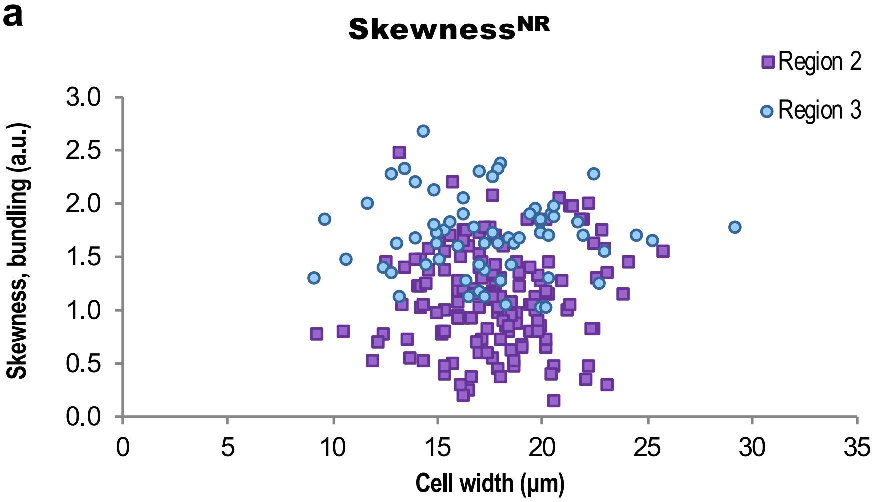
SkewnessNR
Region 2
Region 3
3.0
2.5
2.0
1.5
1.0
0.5
0.0
Skewness, bundling (a.u.)
0
5
10
15
20
25
30
35
Cell width (µm)

b

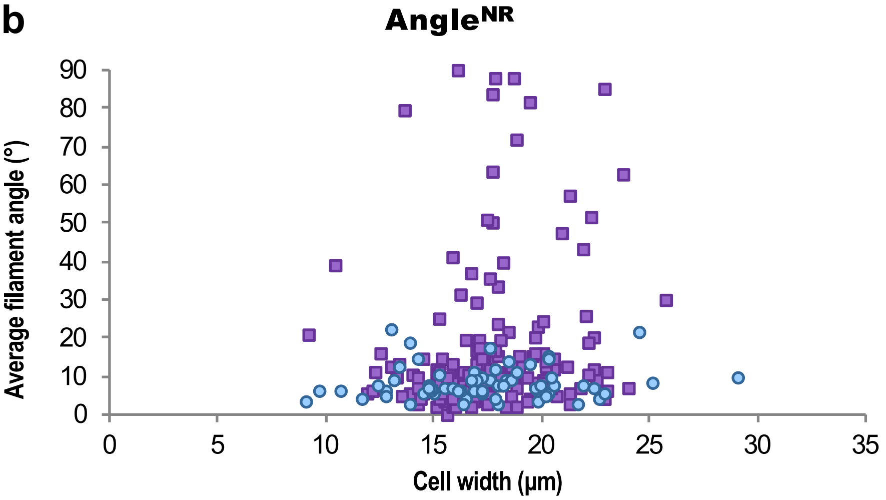
AngleNR
90
80
70
60
50
40
30
20
10
0
Average filament angle (°)
0
5
10
15
20
25
30
35
Cell width (µm)

c

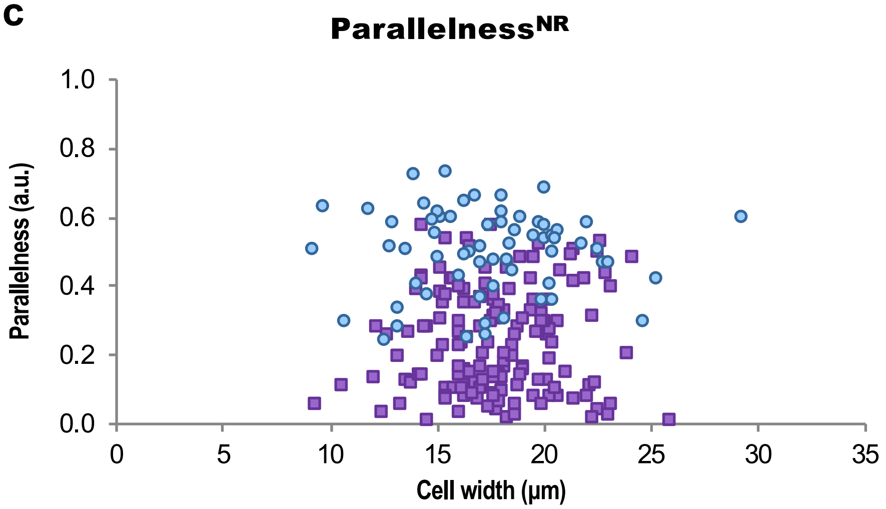
ParallelnessNR
1.0
0.8
0.6
0.4
0.2
0.0
Parallelness (a.u.)
0
5
10
15
20
25
30
35
Cell width (µm)

Fig. S3 Actin filament arrays are predictive of cell length. **(a)** to **(d)** Quantification of individual actin architecture or orientation metrics in *Arabidopsis thaliana* root epidermal cells plotted with respect to corresponding cell length in the elongation zone (Regions 2 and 3), shown with R^2 values (linear fit determined in Excel) for reference only. These graphs are the same as **Fig. 1** but are not shaded by region. Since it is not possible to know whether cell length or the aspects of actin organization is the independent variable, linear regression analysis is not an appropriate statistical metric on which to base strong conclusions. N = 60–150 cells per region from 20 roots. A.u., arbitrary units. NR, no predictive relationship; ***, $p \leq 0.0001$, Bivariate fit/ANOVA for all data points for each parameter. Results are from one experiment.

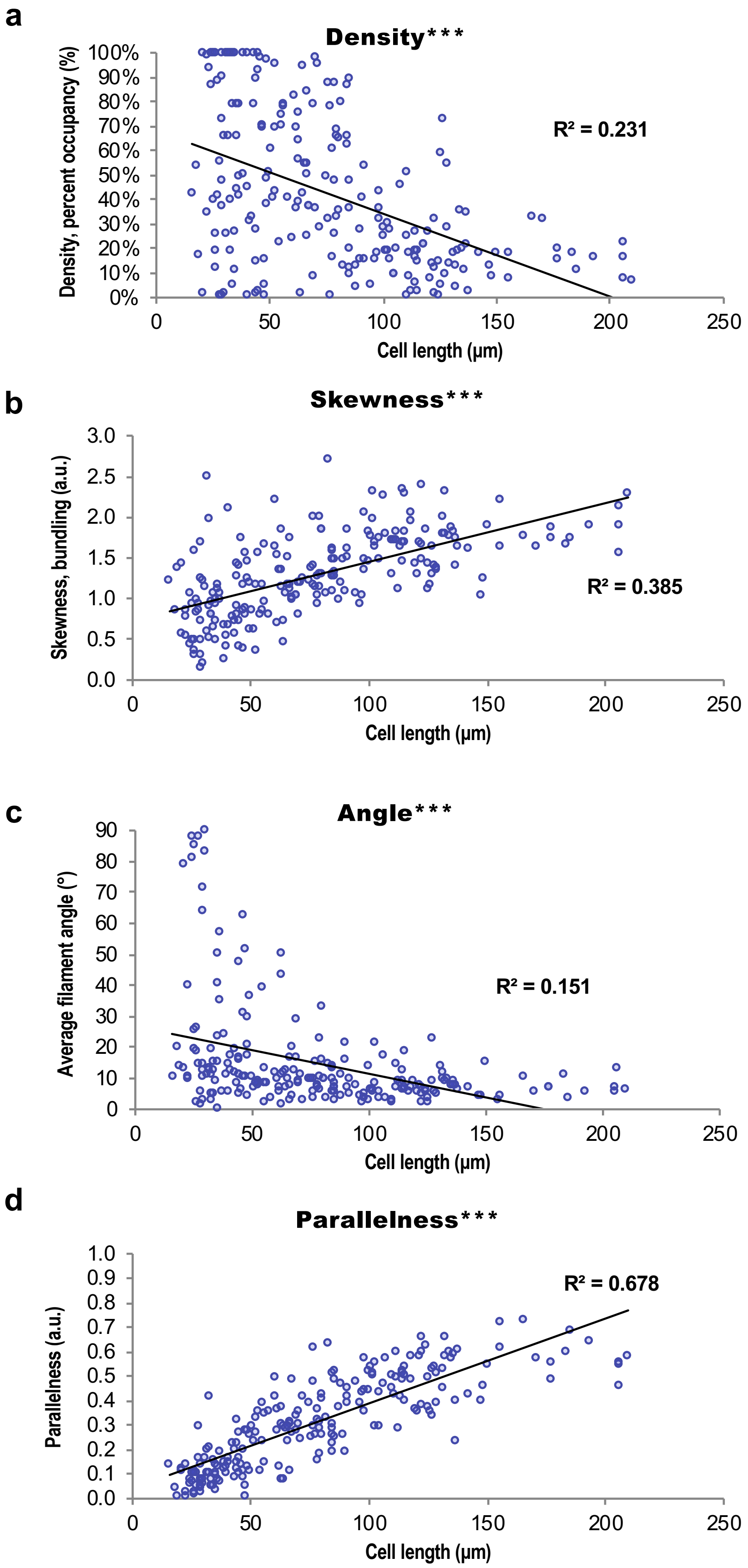
a
Density***
Density, percent occupancy (%)
100%
90%
80%
70%
60%
50%
40%
30%
20%
10%
0%
0
50
100
150
200
250
Cell length (µm)
R² = 0.231
b
Skewness***
Skewness, bundling (a.u.)
3.0
2.5
2.0
1.5
1.0
0.5
0.0
0
50
100
150
200
250
Cell length (µm)
R² = 0.385
c
Angle***
Average filament angle (°)
90
80
70
60
50
40
30
20
10
0
0
50
100
150
200
250
Cell length (µm)
R² = 0.151
d
Parallelness***
Parallelness (a.u.)
1.0
0.9
0.8
0.7
0.6
0.5
0.4
0.3
0.2
0.1
0.0
0
50
100
150
200
250
Cell length (µm)
R² = 0.678

Fig. S4 Actin arrays in *Arabidopsis thaliana* root Region 3 are more dynamic than in Region 2. Filament arrays in short cells (purple squares) exhibited less overall dynamicity (i.e., short cells showed a slower decrease in similarity between pixel intensities and location between time intervals) compared with filament arrays of long cells (blue circles). 100-s timelapse movies were collected from short and long cells in the same 30 roots. N = 80 (Region 3) to 148 (Region 2) cells from the same 30 plants as **Fig. 2** and **Table 1**. Error bars represent ± one standard error. ***, $F \leq 0.001$, oneway ANOVA.

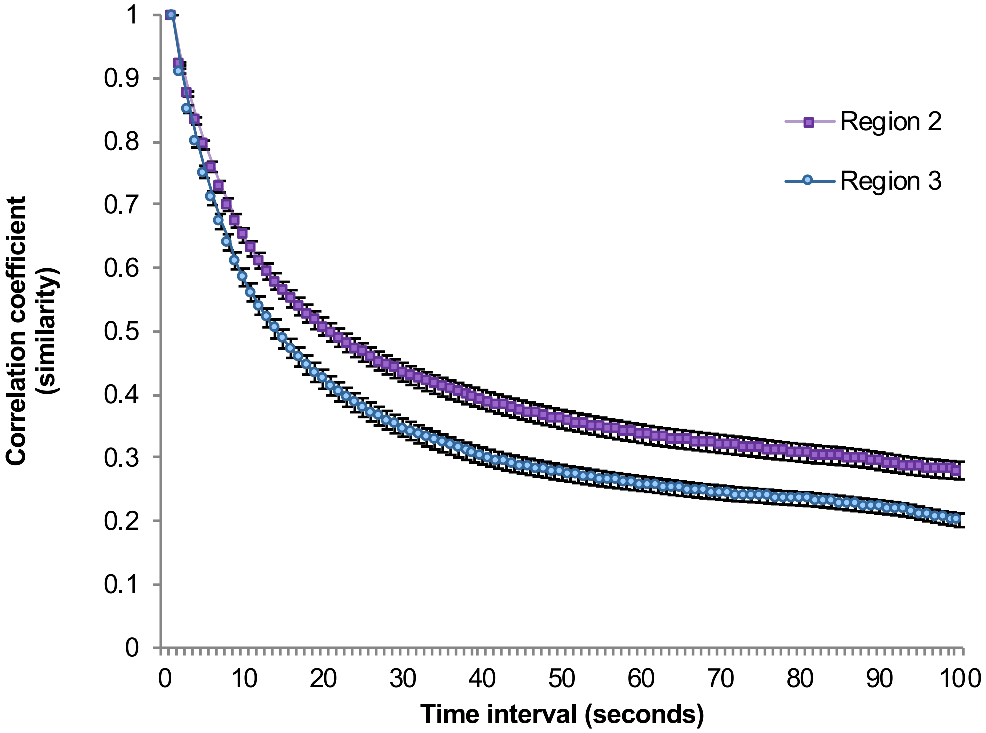

Fig. S5 Short-term IAA treatments induce dose-dependent changes in actin filament organization. **(a)** to **(d)** Quantification of actin orientation in *Arabidopsis thaliana* root epidermal cells: indole-3-acetic acid (IAA) triggered a dose-dependent decrease in average filament angle **(a)** and **(c)** and increase in parallelness **(b)** and **(d)**. Region 2 measurements are shown in **(a)** and **(b)**; Region 3 in **(d)** and **(e)**. The dose-dependency was more pronounced in Region 3. Cells whose lengths fell between 85 and 94 μm were counted in both regions. N = 8–12 cells per region per root from at least 10 roots per treatment. Error bars represent ± one standard error; a.u., arbitrary units. Different letters indicate statistically significant differences, oneway ANOVA, compared with Tukey-Kramer HSD in JMP. All IAA experiments were performed and analyzed double blind.

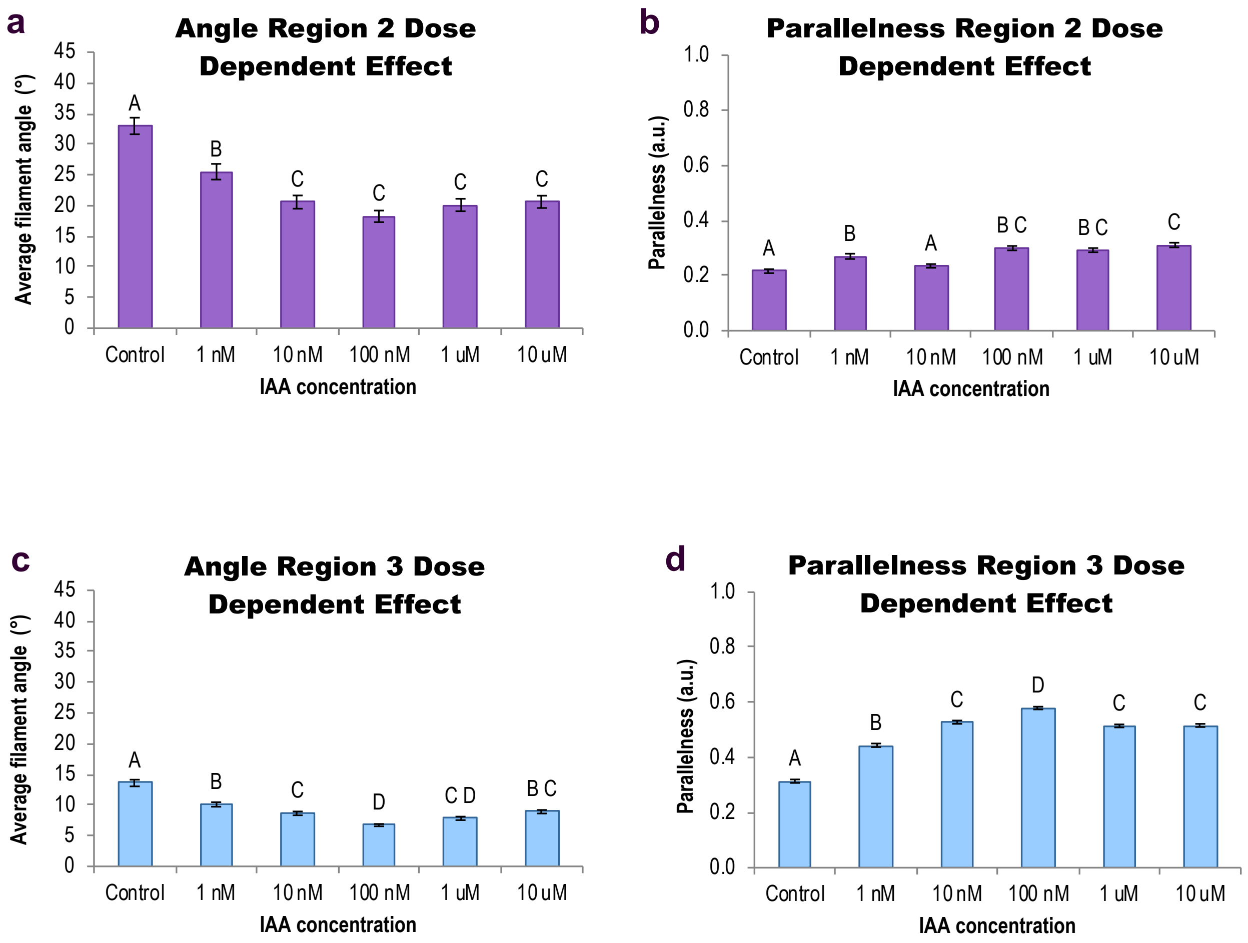

Fig. S6 Short-term IAA treatments induce a time-dependent increase in actin filament density and longitudinal orientation. **(a)** Representative VAEM images of GFP-fABD2–labeled actin in *Arabidopsis thaliana* epidermal cells from Region 2 (left) and Region 3 (right). Scale bar, 10 µm. **(b)** to **(e)** Quantification of actin architecture in Regions 2 and 3: 10 nM indole-3-acetic acid (IAA; dotted lines) triggered time-dependent increased actin filament density **(b)** and **(c)** and decreased skewness **(d)** and **(e)** compared to control (unbroken lines). Region 2 measurements **(b)**, **(d)**, **(f)**, and **(h)** are shown in purple; Region 3 **(c)**, **(e)**, **(g)**, and **(i)** in blue. **(f)** to **(i)** Quantification of actin orientation in Regions 2 and 3: after 10 nM IAA treatments, actin in both regions appeared more 'organized', with lower average filament angle **(f)** and **(g)** relative to the longitudinal axis of the cell and filaments generally more parallel to each other **(h)** and **(i)**. These responses were roughly time-dependent, with a peak in parallel longitudinality occurring after 20–30 min of treatment. N = 7 roots per treatment per timepoint (≥ 10 cells from Region 2 and *c*. 4–9 cells from Region 3 from each root). Error bars represent ± one standard error. ND, no statistical differences; *, $p \leq 0.05$; **, $p \leq 0.01$; ***, $p \leq 0.001$, Student's t-test of IAA (dotted line) vs. control (unbroken line) on that region at that timepoint. Results are from one representative experiment of two similar experiments with similar results. All IAA experiments were performed and analyzed double blind.

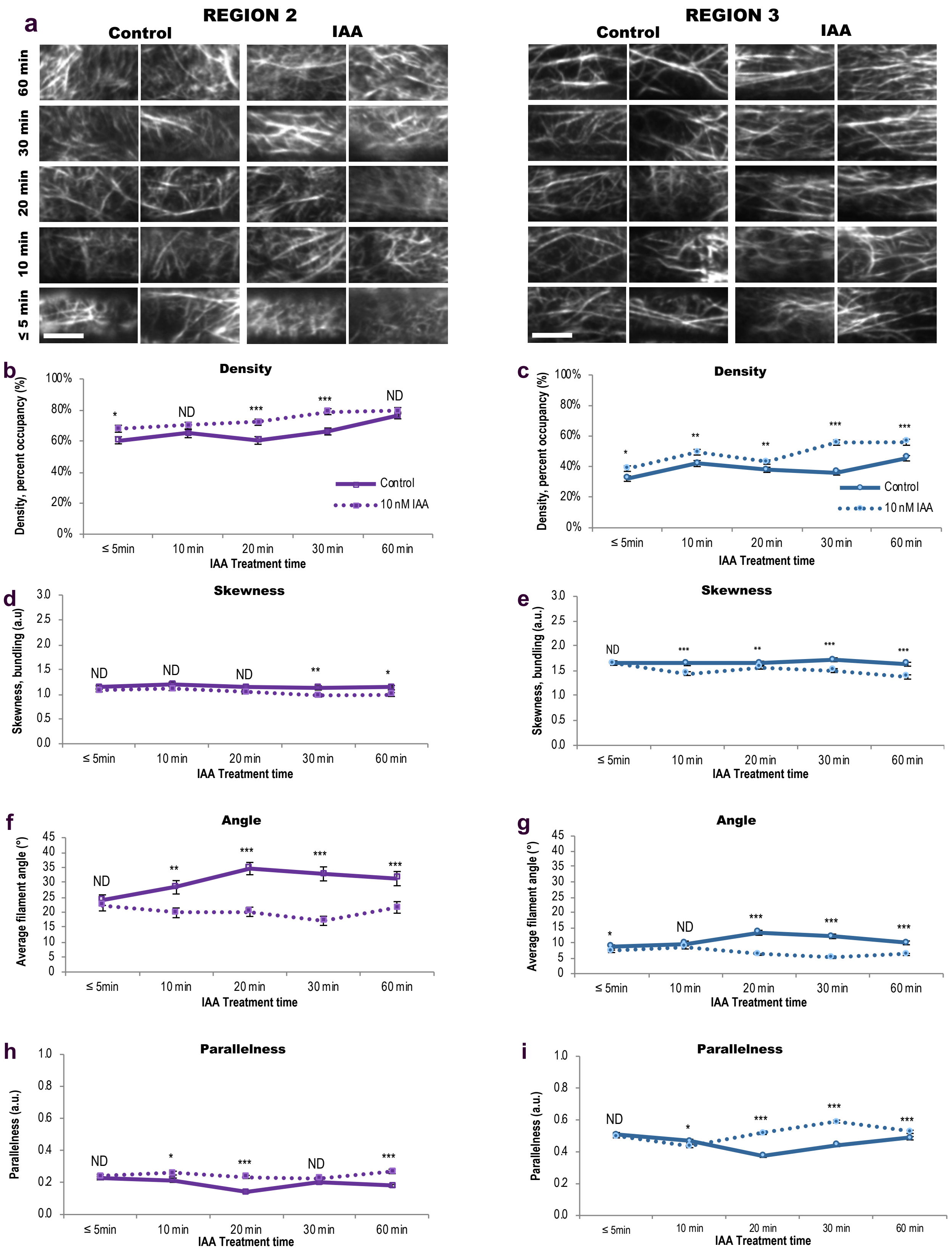

a
REGION 2
Control
IAA
REGION 3
Control
IAA
60 min
30 min
20 min
10 min
≤ 5 min
b
Density
Density, percent occupancy (%)
Control
10 nM IAA
IAA Treatment time
c
Density
d
Skewness
Skewness, bundling (a.u)
e
Skewness
f
Angle
Average filament angle (°)
g
Angle
h
Parallelness
Parallelness (a.u.)
i
Parallelness
ND
≤ 5min
10 min
20 min
30 min
60 min

Fig. S7 Actin filament organization plotted with respect to corresponding cell length in WS and *aux1-100*. **(a)** to **(d)** Quantification of individual actin architecture or orientation metrics plotted with respect to corresponding cell length in WS (blue squares) and *aux1-100* (green circles) in *Arabidopsis thaliana* roots. **(e)** to **(h)** Quantification of WS individual actin architecture or orientation metrics after treatment with control (blue squares), indole-3-acetic acid (IAA; blue triangles), and 1-naphthylacetic acid (NAA; (blue diamonds), plotted with respect to corresponding cell length. **(i)** to **(l)** Quantification of *aux1-100* individual actin architecture or orientation metrics after treatment with control (green circles), IAA (green triangles), and NAA (green diamonds), plotted with respect to corresponding cell length. These graphs are scatterplots representing the same dataset as **Fig. 5**; actin measurements were quantified on a per-cell basis and each data point represents a single cell's actin array plotted against its length. A.u., arbitrary units.

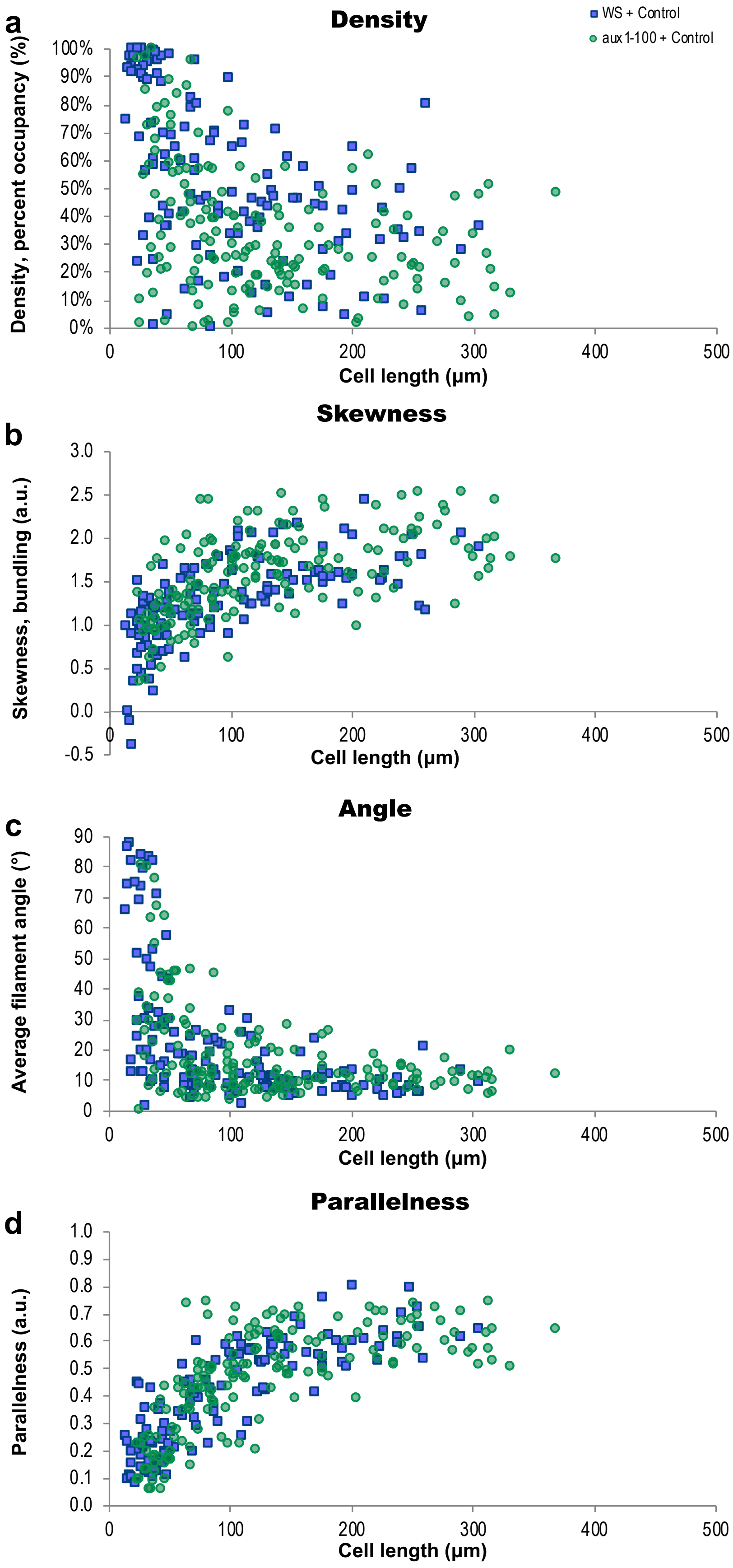
a
Density
WS + Control
aux1-100 + Control
Density, percent occupancy (%)
100%
90%
80%
70%
60%
50%
40%
30%
20%
10%
0%
0
100
200
300
400
500
Cell length (µm)
b
Skewness
Skewness, bundling (a.u.)
3.0
2.5
2.0
1.5
1.0
0.5
0.0
-0.5
0
100
200
300
400
500
Cell length (µm)
c
Angle
Average filament angle (°)
90
80
70
60
50
40
30
20
10
0
0
100
200
300
400
500
Cell length (µm)
d
Parallelness
Parallelness (a.u.)
1.0
0.9
0.8
0.7
0.6
0.5
0.4
0.3
0.2
0.1
0.0
0
100
200
300
400
500
Cell length (µm)

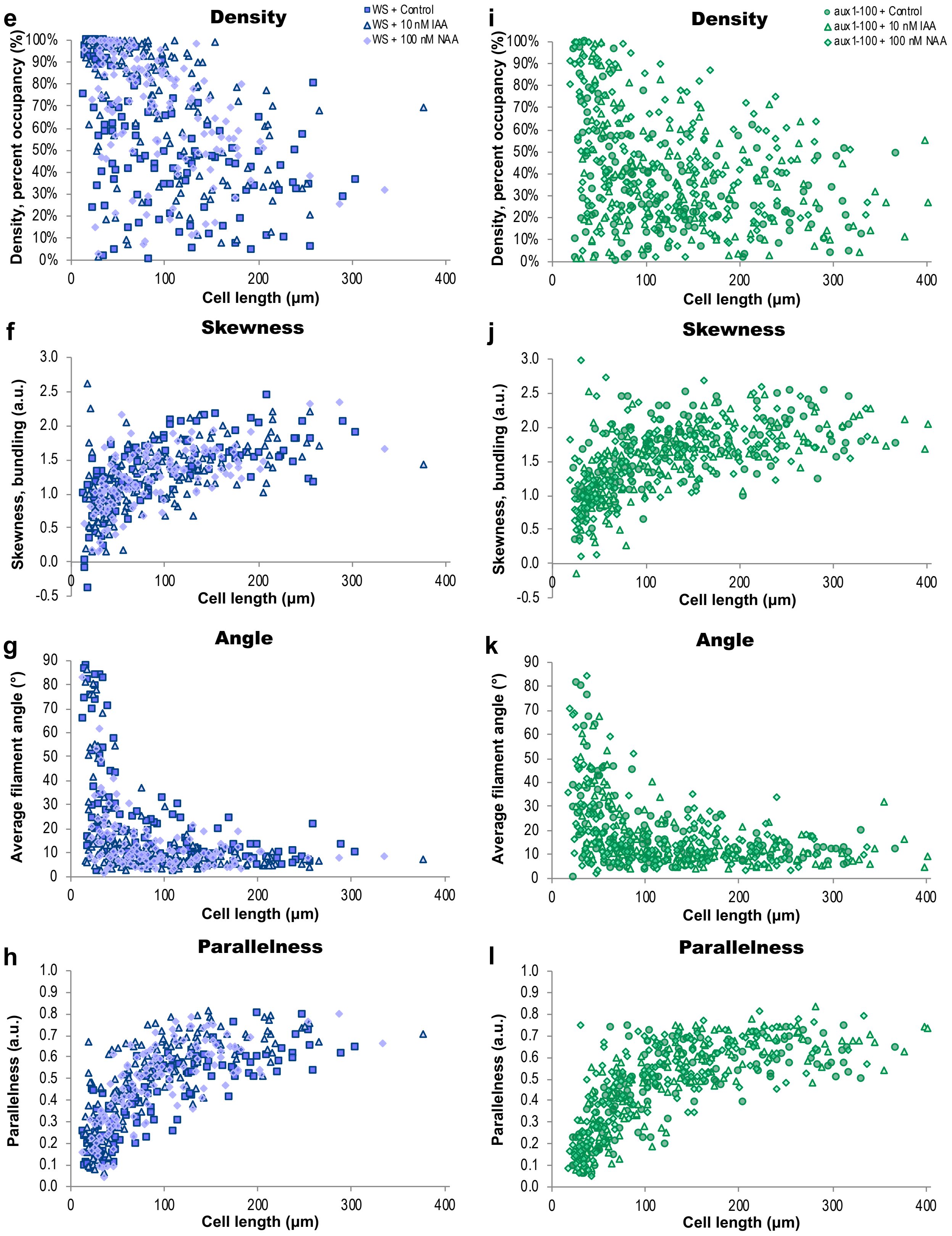

e
Density
WS + Control
WS + 10 nM IAA
WS + 100 nM NAA
Density, percent occupancy (%)
Cell length (µm)
i
Density
aux1-100 + Control
aux1-100 + 10 nM IAA
aux1-100 + 100 nM NAA
Density, percent occupancy (%)
Cell length (µm)
f
Skewness
Skewness, bundling (a.u.)
Cell length (µm)
j
Skewness
Skewness, bundling (a.u.)
Cell length (µm)
g
Angle
Average filament angle (°)
Cell length (µm)
k
Angle
Average filament angle (°)
Cell length (µm)
h
Parallelness
Parallelness (a.u.)
Cell length (µm)
l
Parallelness
Parallelness (a.u.)
Cell length (µm)

Fig. S8 Actin organization in *aux1-22* fails to respond to short-term IAA treatments but partially responds to the membrane-permeable auxin NAA. **(a)** to **(c)** Representative VAEM images of GFP-fABD2–labeled actin in *Arabidopsis thaliana* epidermal cells from wildtype (Col-0) and *aux1-22*, treated for 20–30 min with control **(a)**, 10 nM indole-3-acetic acid (IAA) **(b)**, or 100 nM 1-naphthylacetic acid (NAA) **(c)**. Scale bar, 5 µm. **(d)** to **(g)** Quantification of actin organization in root epidermal cells. Both IAA and NAA failed to trigger an increase in actin filament density **(d)** and decrease in skewness **(e)** in *aux1-22* but actin density in wildtype cells increased in response to both IAA and NAA and skewness decreased with both auxin treatments. Wildtype response is shown in blue and *aux1-22* in green; control, solid; 10 nM IAA, dots; 100 nM NAA, stripes. After IAA treatment, actin arrays in wildtype plants were more 'organized,' with lower average filament angle **(f)** relative to the longitudinal axis of the cell and filaments generally more parallel to each other **(g)**. NAA triggered the increase in actin density in wildtype plants, but had no effect on angle or parallelness when measured on a per-cell basis. Average actin filament angle and parallelness in *aux1-22* failed to reorganize in response to IAA and only average filament angle decreased (and no increase in parallelness) with the membrane-permeable auxin NAA. N = 5–38 cells per root; 10 roots per genotype per treatment. Error bars represent ± one standard error; a.u., arbitrary units. Different letters indicate statistically significant differences, oneway ANOVA, compared with Tukey-Kramer HSD in JMP (see Methods for more information). Actin measurements were quantified on a per-cell basis; see Methods for description and Supplemental for scatter plots. Results are from one experiment. All auxin experiments were performed and analyzed double blind.

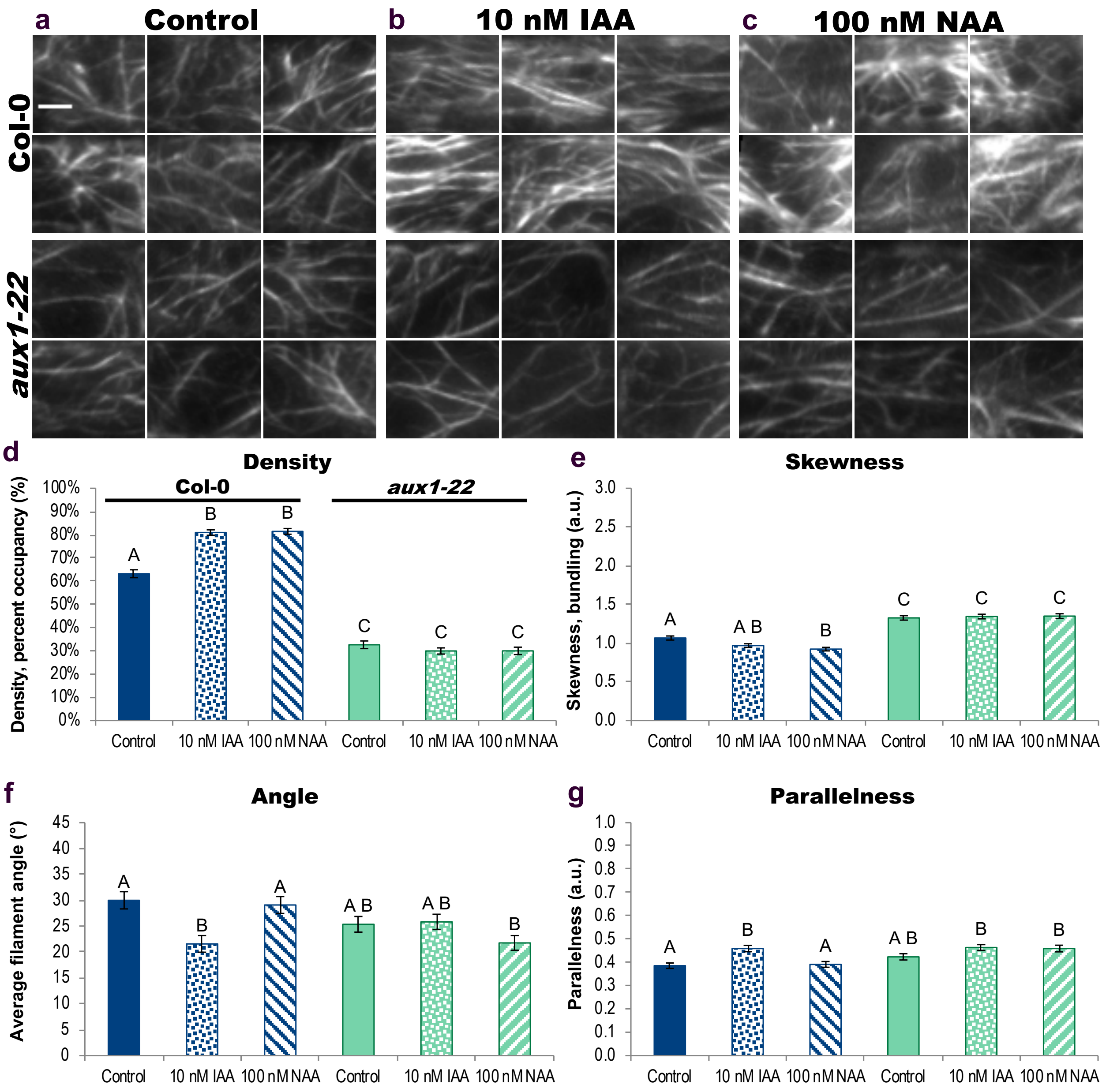

a
Control
b
10 nM IAA
c
100 nM NAA
Col-0
aux1-22
d
Density
Col-0
aux1-22
Density, percent occupancy (%)
100%
90%
80%
70%
60%
50%
40%
30%
20%
10%
0%
A
B
B
C
C
C
Control
10 nM IAA
100 nM NAA
Control
10 nM IAA
100 nM NAA
e
Skewness
Skewness, bundling (a.u.)
3.0
2.5
2.0
1.5
1.0
0.5
0.0
A
A B
B
C
C
C
Control
10 nM IAA
100 nM NAA
Control
10 nM IAA
100 nM NAA
f
Angle
Average filament angle (°)
45
40
35
30
25
20
15
10
5
0
A
B
A
A B
A B
B
Control
10 nM IAA
100 nM NAA
Control
10 nM IAA
100 nM NAA
g
Parallelness
Parallelness (a.u.)
1.0
0.9
0.8
0.7
0.6
0.5
0.4
0.3
0.2
0.1
0.0
A
B
A
A B
B
B
Control
10 nM IAA
100 nM NAA
Control
10 nM IAA
100 nM NAA

Fig. S9 Actin filament organization plotted with respect to corresponding cell length in Col-0 and *aux1-22*. **(a)** to **(d)** Quantification of individual actin architecture or orientation metrics plotted with respect to corresponding cell length in Col-0 (blue squares) and *aux1-22* (green circles) in *Arabidopsis thaliana* roots*.* **(e)** to **(h)** Quantification of Col-0 individual actin architecture or orientation metrics after treatment with control (blue squares), indole-3-acetic acid (IAA; blue triangles), and 1-naphthylacetic acid (NAA; blue diamonds), plotted with respect to corresponding cell length*.* **(i)** to **(l)** Quantification of *aux1-22* individual actin architecture or orientation metrics after treatment with control (green circles), IAA (green triangles), and NAA (green diamonds), plotted with respect to corresponding cell length*.* These graphs are scatterplots representing the same dataset as Supporting Information Fig. **S8**; actin measurements were quantified on a per-cell basis and each data point represents a single cell's actin array plotted against its length. A.u., arbitrary units.

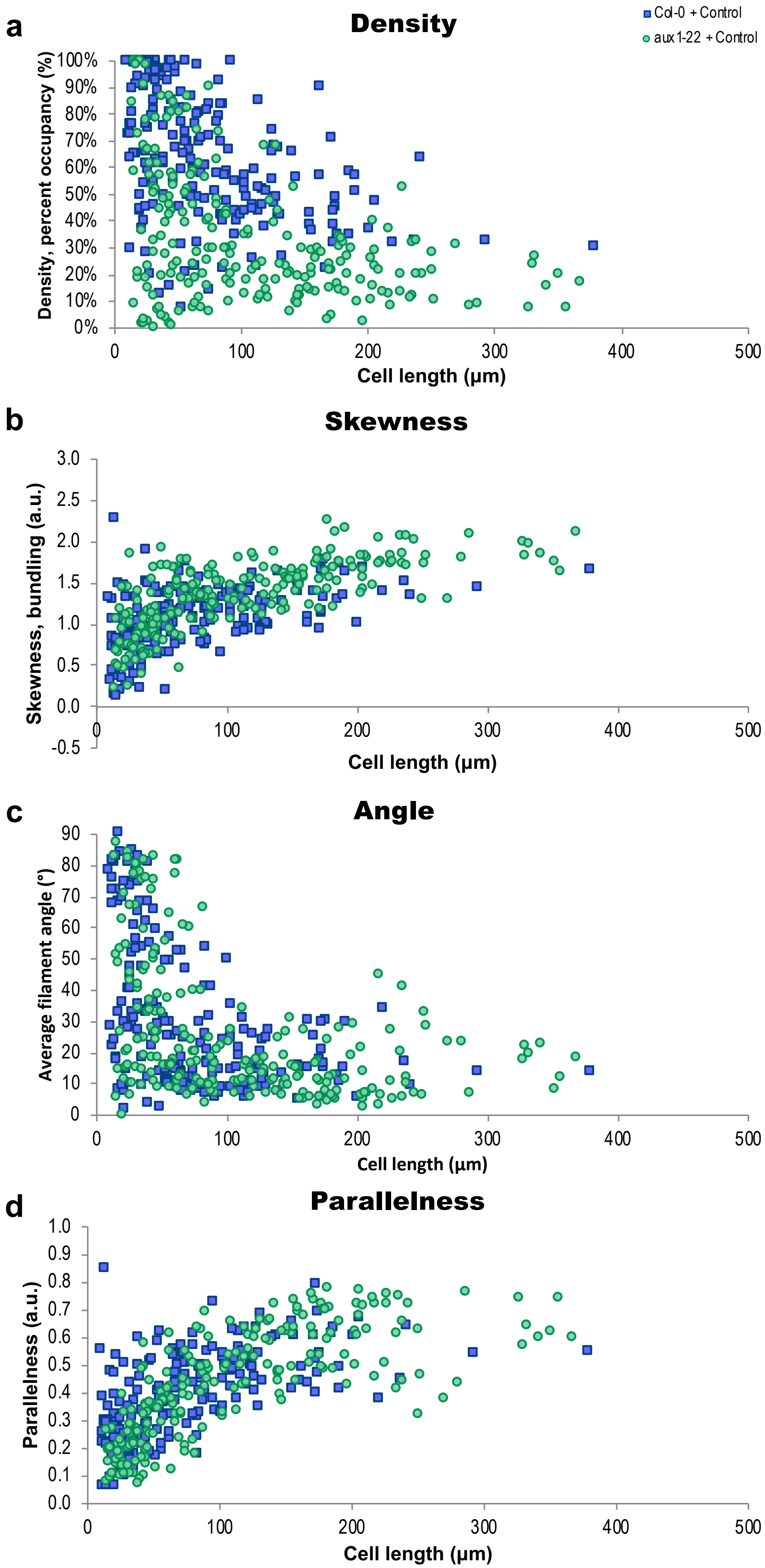

a
Density
Col-0 + Control
aux1-22 + Control
Density, percent occupancy (%)
100%
90%
80%
70%
60%
50%
40%
30%
20%
10%
0%
0
100
200
300
400
500
Cell length (µm)
b
Skewness
Skewness, bundling (a.u.)
3.0
2.5
2.0
1.5
1.0
0.5
0.0
-0.5
Cell length (µm)
c
Angle
Average filament angle (°)
90
80
70
60
50
40
30
20
10
0
Cell length (µm)
d
Parallelness
Parallelness (a.u.)
1.0
0.9
0.8
0.7
0.6
0.5
0.4
0.3
0.2
0.1
0.0
Cell length (µm)

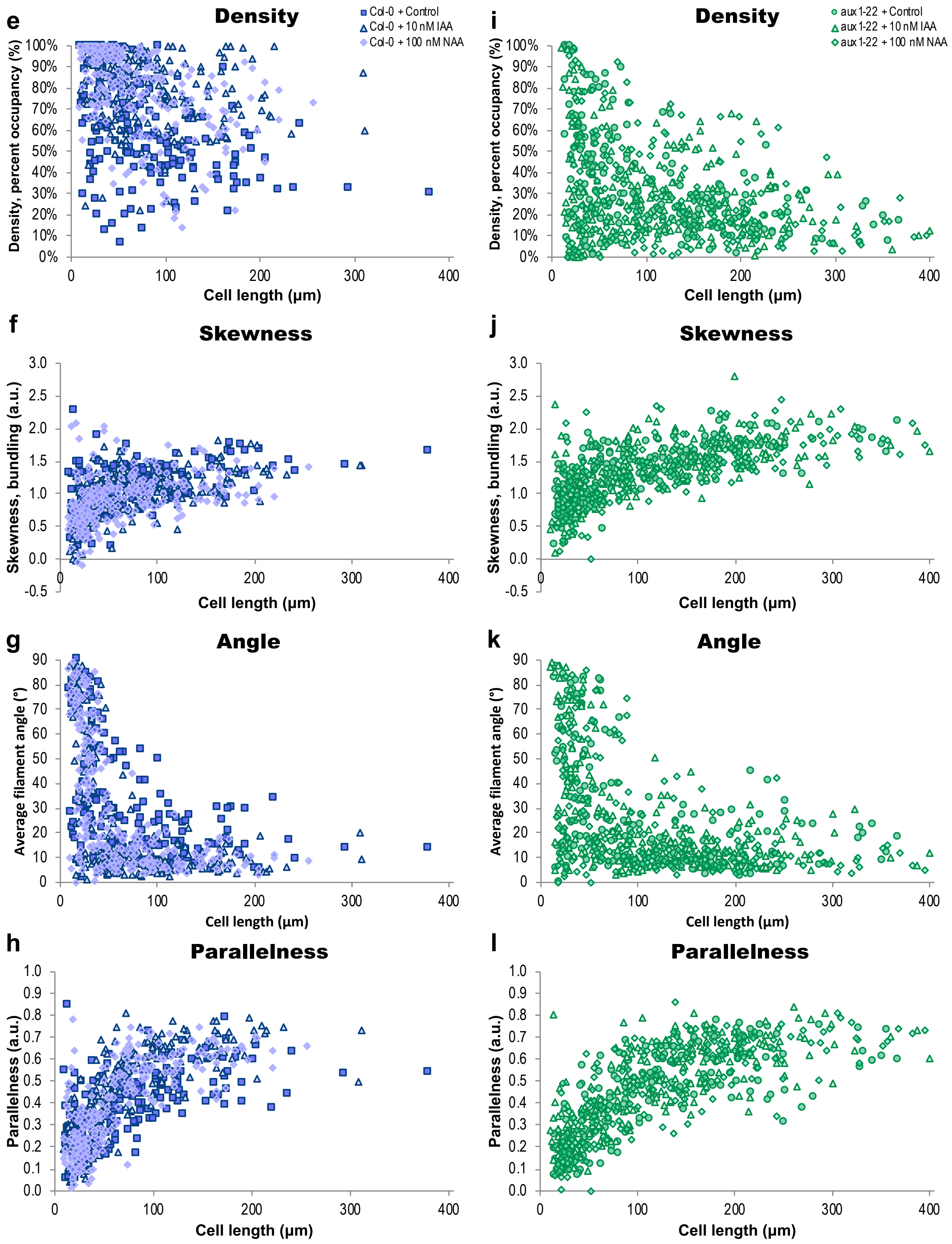

e
Density
Col-0 + Control
Col-0 + 10 nM IAA
Col-0 + 100 nM NAA
Density, percent occupancy (%)
Cell length (µm)
i
Density
aux1-22 + Control
aux1-22 + 10 nM IAA
aux1-22 + 100 nM NAA
Density, percent occupancy (%)
Cell length (µm)
f
Skewness
Skewness, bundling (a.u.)
Cell length (µm)
j
Skewness
Skewness, bundling (a.u.)
Cell length (µm)
g
Angle
Average filament angle (°)
Cell length (µm)
k
Angle
Average filament angle (°)
Cell length (µm)
h
Parallelness
Parallelness (a.u.)
Cell length (µm)
l
Parallelness
Parallelness (a.u.)
Cell length (µm)

Fig. S10 Actin organization in wildtype WS fails to respond to short-term auxin treatments at high pH. **(a)** to **(d)** Quantification of actin organization in WS root epidermal cells treated for 20–30 min with control, 10 nM indole-3-acetic acid (IAA), or 100 nM 1-naphthylacetic acid (NAA). Control, solid; 10 nM IAA, dots; 100 nM NAA, stripes. These results should be compared with WS actin response to IAA and NAA at pH ≈ 5.8 in Fig. **5**. Fig. **5** illustrates the substantial actin reorganization that occurs in response to auxins at a biologically relevant pH and which is lacking here at pH > 10. N = crops from the entire root elongation zone of 10 roots per treatment. Error bars represent ± one standard error; a.u., arbitrary units. Actin measurements were quantified overall (i.e., **not** on a per-cell basis), similar to Figs. **1**, **3**, **S1**, **S5**, and **S6**). Results are from one experiment. All auxin experiments were performed and analyzed double blind. To obtain high pH, auxins were dissolved in 1 M NaOH and equivalent NaOH was added to control solution.

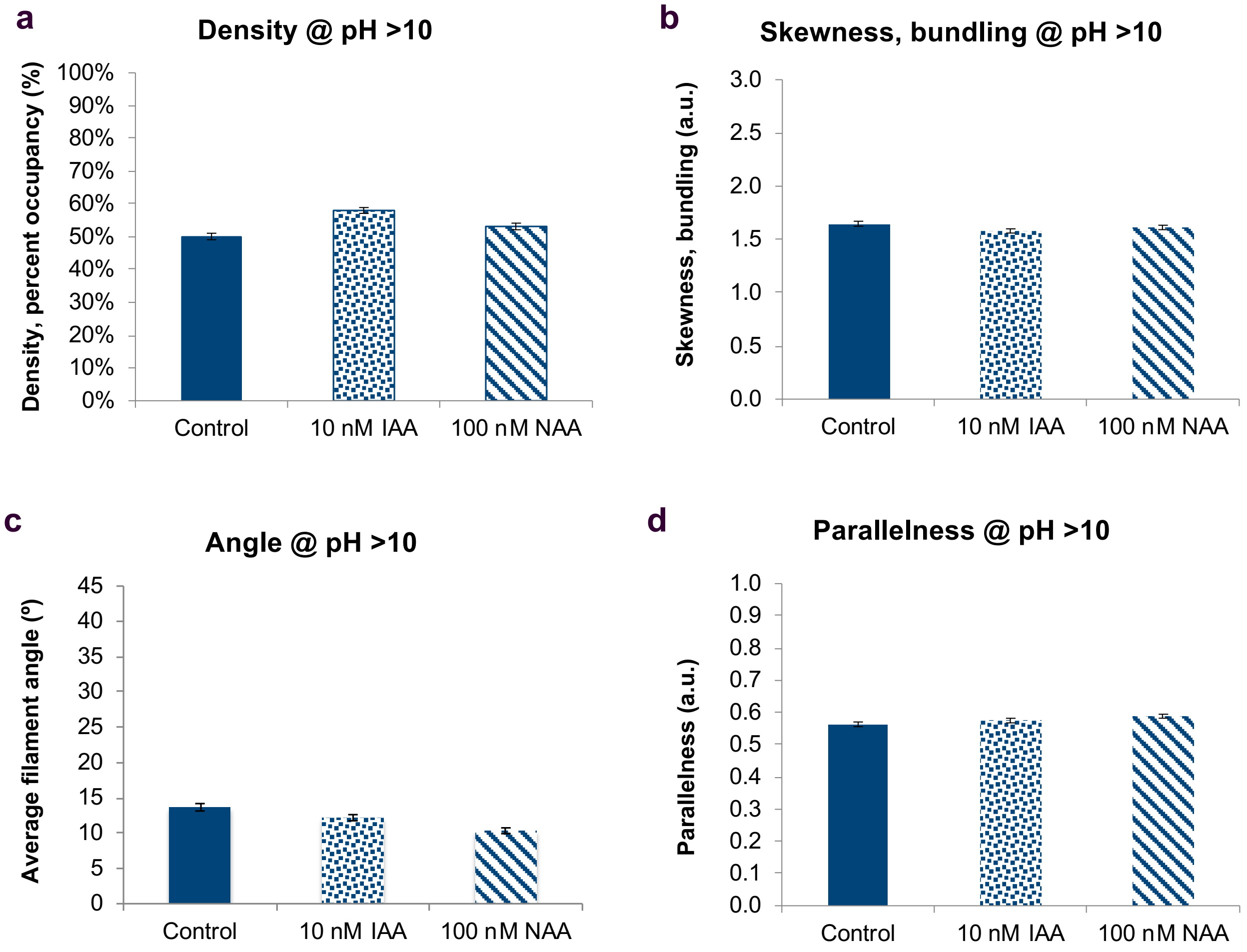

Fig. S11 Epidermal cell elongation rate increases as cells undergo axial expansion in the *Arabidopsis thaliana* root elongation zone. **(a)** To **(c)** Maximum projection of longterm, lightsheet fluorescence microscope (LSFM) images of root epidermal cells from the root cap, transition, and elongation zones at T = 0 **(a)**; T = 5 h **(b)**; and T = 9 h **(c)**. LSFM enabled measurement of individual epidermal cell lengths over a 10 h period. Blue numbers labeling cells in **(a)** to **(c)** correspond to cell length plots in **(d)**. Cell 13 was not discernable until Frame 3, but its approximate location is marked in parentheses at T0. Some labeled cells grew out of the field of view by the end of imaging. Scale bar, 50 µm. **(d)** Individual cell lengths plotted over time from 27 epidermal cells in a timelapse movie (Video **S1**) of a single Col-0 root. Line colors indicate visual region assignment at end of imaging: Purple, cell started and remained in Region 2; Dark blue, cell started in Region 2 and ended in Region 3; light blue, cell started in Region 3 and ended in Region 3. Pink triangles, timepoint of root hair (RH) emergence on trichoblasts. Note that many epidermal cells undergo a transition from modest or slow growth to rapid cell axial cell expansion at a size of 20–30 µm. Root hair emergence from trichoblasts occurred during or after the rapid growth phase, typically at a size of 60–120 µm. At cell lengths greater than 90–100 µm, most epidermal cells transitioned to slow or no axial expansion. This root was imaged every 15 min for a total of 10 h (40 total frames) in media (0.6% agar, sucrose-free 1⁄2 MS) inside a capillary tube on the Bruker/Luxendo MuVi platform; final magnification of 22.2×. Results are from one experiment. **NOTE:** Brightness and contrast were enhanced in the longterm projection of Figs. **S11a–c** to better show the cell outlines and actin structures.

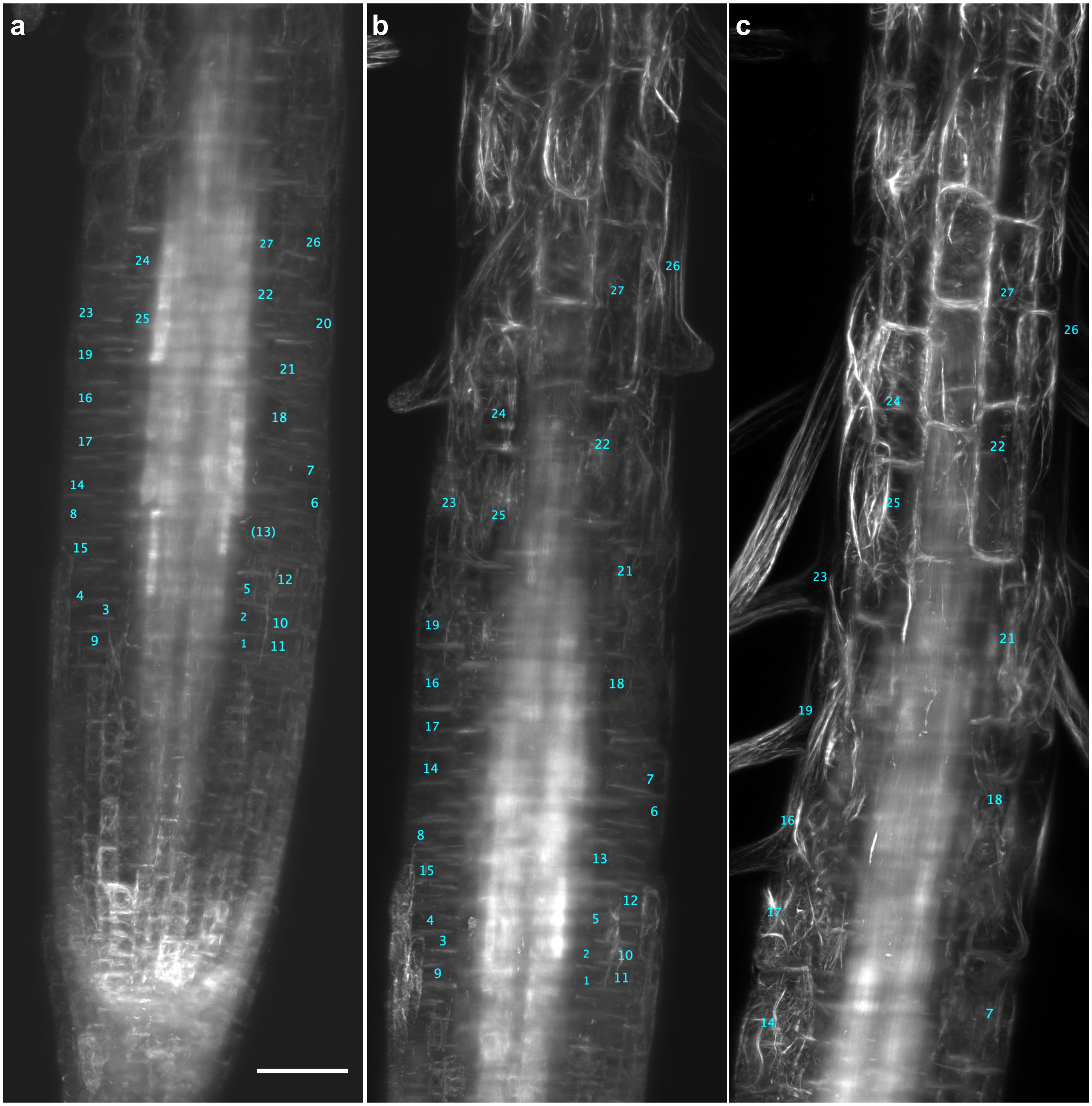

a
27 26
24
22
23
25
20
19
21
16
18
17
7
14
6
8
(13)
15
12
5
4
3
2
10
9
1
11
b
26
27
24
22
23
25
21
19
16
18
17
14
7
6
8
13
15
12
4
5
3
2
10
9
1
11
c
27
26
24
22
25
23
21
19
18
16
17
14
7

d

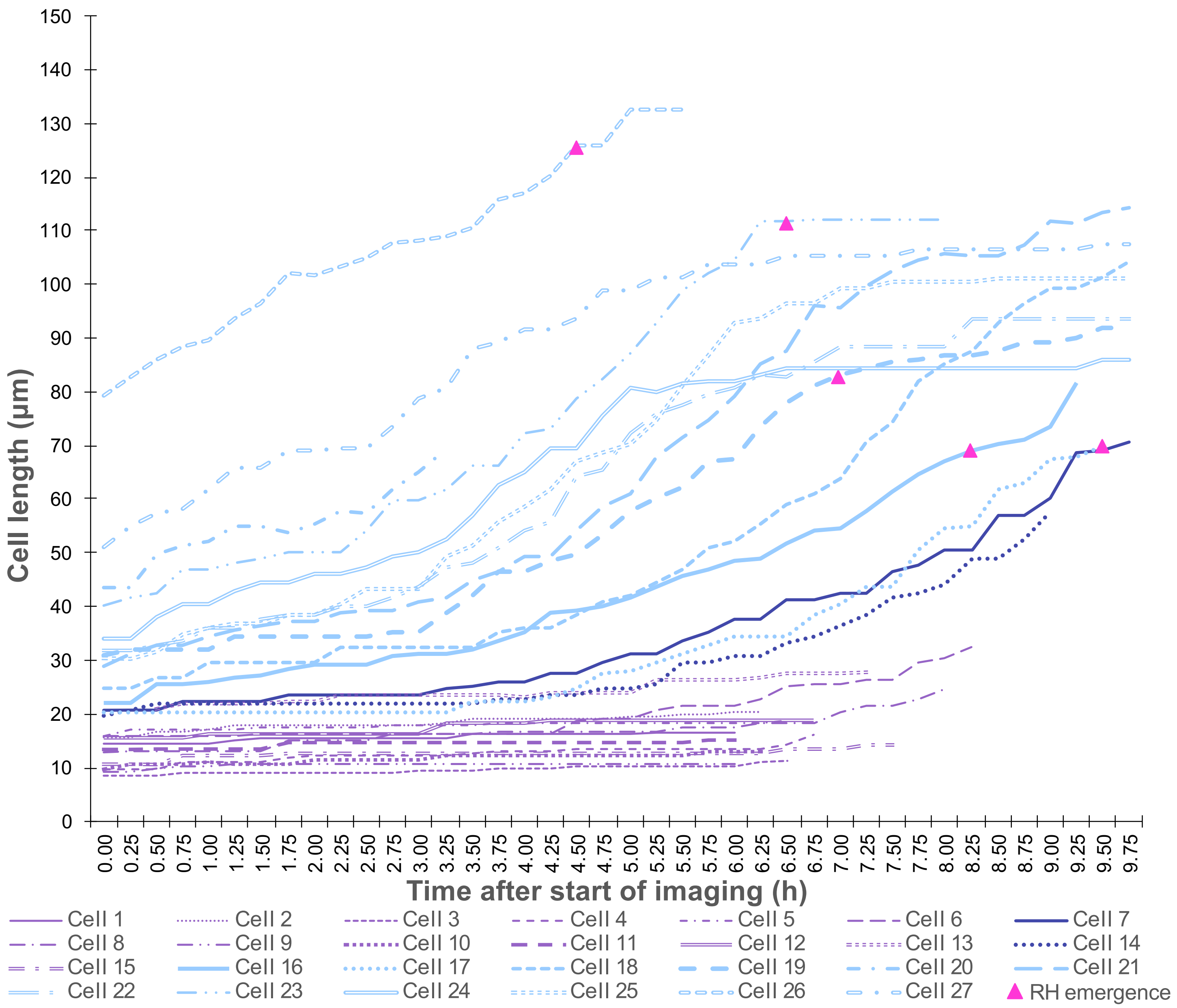

Cell length (µm)
150
140
130
120
110
100
90
80
70
60
50
40
30
20
10
0
0.00 0.25 0.50 0.75 1.00 1.25 1.50 1.75 2.00 2.25 2.50 2.75 3.00 3.25 3.50 3.75 4.00 4.25 4.50 4.75 5.00 5.25 5.50 5.75 6.00 6.25 6.50 6.75 7.00 7.25 7.50 7.75 8.00 8.25 8.50 8.75 9.00 9.25 9.50 9.75
Time after start of imaging (h)
Cell 1
Cell 2
Cell 3
Cell 4
Cell 5
Cell 6
Cell 7
Cell 8
Cell 9
Cell 10
Cell 11
Cell 12
Cell 13
Cell 14
Cell 15
Cell 16
Cell 17
Cell 18
Cell 19
Cell 20
Cell 21
Cell 22
Cell 23
Cell 24
Cell 25
Cell 26
Cell 27
RH emergence

Table S1 Eigenvectors for principal component analysis of cell size vs. actin parameters in *Arabidopsis thaliana* ecotype Col-0.

Item	Prin1	Prin2	Prin3	Prin4	Prin5	Prin6
Cell Length	0.527	0.093	0.048	0.044	0.609	-0.582
Cell Width	-0.032	0.876	-0.392	-0.270	-0.069	-0.014
Density	-0.360	0.096	-0.361	0.813	0.266	-0.000
Skewness	0.440	0.238	0.273	0.495	-0.634	-0.168
Angle	-0.325	0.392	0.797	0.072	0.284	0.137
Parallelness	0.542	0.067	-0.053	0.122	0.266	0.784

Table S1 Entries show the correlation between each attribute (i.e., cell length, cell width, etc.) and a principal component. Text shading emphasizes the magnitude of that correlation.

Table S2 Eigenvalues for principal component analysis of cell size vs. actin parameters in *Arabidopsis thaliana* ecotype Col-0.

Number	Eigenvalue	Percent	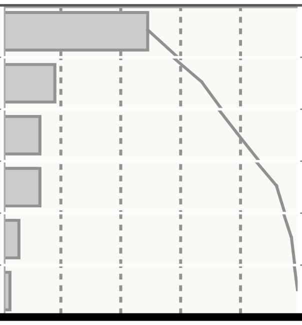	Cum. Percent
1	2.9322	48.871		48.871
2	1.0720	17.867		66.737
3	0.7749	12.914		79.652
4	0.7373	12.288		91.940
5	0.3217	5.362		97.302
6	0.1619	2.698		100.000

Table S2 Bars indicate percent of variance that each principal component (in Table **S1**) explains in the sample. Solid line indicates cumulative percentage of variance accounted for; dashed lines mark quintiles.

Table S3 Eigenvectors for principal component analysis of cell size vs. actin parameters in *Arabidopsis thaliana* ecotype WS.

Item	Prin1	Prin2	Prin3	Prin4	Prin5	Prin6
WS Cell Length	0.53137	0.13361	0.01256	0.10846	0.34014	0.75643
WS Cell Width	-0.02484	0.91905	0.18593	-0.33084	0.00615	-0.10329
WS Density	-0.33979	0.06901	0.72268	0.57098	-0.06879	0.16357
WS Skewness	0.48014	0.14062	-0.09248	0.30058	-0.80565	-0.04142
WS Angle	-0.32848	0.33435	-0.64176	0.58653	0.15359	0.02918
WS Parallelness	0.51297	0.03429	0.15030	0.34408	0.45483	-0.62276

Table S3 Entries show the correlation between each attribute (i.e., cell length, cell width, etc.) and a principal component. Text shading emphasizes the magnitude of that correlation.

Table S4 Eigenvalues for principal component analysis of cell size vs. actin parameters in *Arabidopsis thaliana* ecotype WS.

Number	Eigenvalue	Percent	Cum. Percent
1	2.9778	49.630	49.630
2	1.0810	18.016	67.646
3	0.9457	15.762	83.408
4	0.4634	7.724	91.132
5	0.3727	6.212	97.344
6	0.1594	2.656	100.000

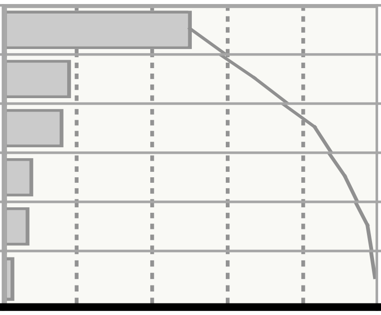

Table S4 Bars indicate percent of variance that each component (in Table **S3**) explains in the sample. Solid line indicates cumulative percentage of variance accounted for; dashed lines mark quintiles.

Table S5 Eigenvectors for principal component analysis of cell size vs. actin parameters in *Arabidopsis thaliana aux1-100.*

Item	Prin1	Prin2	Prin3	Prin4	Prin5	Prin6
aux1-100 Cell Length	0.47215	-0.16402	0.13255	0.22737	0.62559	-0.53810
aux1-100 Cell Width	0.21551	0.94029	-0.12556	-0.17605	0.14714	-0.03177
aux1-100 Density	-0.39119	0.11131	-0.60851	0.66161	0.10752	-0.12251
aux1-100 Skewness	0.45730	0.10303	0.14257	0.45766	-0.70733	-0.22399
aux1-100 Angle	-0.35628	0.24170	0.74402	0.43920	0.17873	0.19035
aux1-100 Parallelness	0.49148	-0.08679	-0.14993	0.27792	0.20778	0.77976

Table S5 Entries show the correlation between each attribute (i.e., cell length, cell width, etc.) and a principal component. Text shading emphasizes the magnitude of that correlation.

Table S6 Eigenvalues for principal component analysis of cell size vs. actin parameters in *Arabidopsis thaliana aux1-100.*

Number	Eigenvalue	Percent		Cum. Percent
1	3.4137	56.895		56.895
2	0.9155	15.258		72.153
3	0.7524	12.539		84.692
4	0.4141	6.901		91.593
5	0.3311	5.519		97.112
6	0.1733	2.888		100.000

Table S6 Bars indicate percent of variance that each component (in Table **S5**) explains in the sample. Solid line indicates cumulative percentage of variance accounted for; dashed lines mark quintiles.

Table S7 Actin organization measurements in *Arabidopsis thaliana* roots after IAA treatments.

Time	Region 2				Region 3			
	Density (%)	Skewness (a.u.)	Angle (°)	Parallelness (a.u)	Density (%)	Skewness (a.u.)	Angle (°)	Parallelness (a.u.)
**≤ 5 min[1]**	67.9 ± 2.24**	1.08 ± 0.03ND	22.2 ± 1.75ND	0.24 ± 0.012ND	38.7 ± 1.80*	1.65 ± 0.04ND	7.3 ± 0.40*	0.50 ± 0.011ND
Control	*60.5 ± 2.29*	*1.13 ± 0.03*	*24.1 ± 1.77*	*0.23 ± 0.011*	*32.2 ± 1.81*	*1.66 ± 0.04*	*8.8 ± 0.59*	*0.51 ± 0.012*
**10 min**	69.9 ± 2.18ND	1.11 ± 0.04ND	19.8 ± 1.63**	0.26 ± 0.012*	49.5 ± 1.85**	1.45 ± 0.04***	8.6 ± 0.55ND	0.44 ± 0.010*
Control	*64.7 ± 2.60*	*1.20 ± 0.05*	*28.4 ± 2.24*	*0.21 ± 0.013*	*42.1 ± 1.96*	*1.64 ± 0.04*	*9.8 ± 0.79*	*0.47 ± 0.012*
**20 min**	72.1 ± 2.00***	1.04 ± 0.04ND	20.2 ± 1.55***	0.23 ± 0.010***	43.0 ± 1.41*	1.56 ± 0.03ND	6.2 ± 0.25***	0.52 ± 0.007***
Control	*60.4 ± 2.47*	*1.13 ± 0.04*	*34.6 ± 2.06*	*0.14 ± 0.007*	*37.9 ± 1.67*	*1.66 ± 0.04*	*13.4 ± 0.81*	*0.37 ± 0.009*
**30 min**	78.9 ± 1.93***	0.98 ± 0.03**	17.1 ± 1.53***	0.22 ± 0.010ND	55.8 ± 1.76***	1.50 ± 0.04***	5.4 ± 0.41***	0.59 ± 0.009***
Control	*66.1 ± 2.24*	*1.13 ± 0.04*	*32.9 ± 2.35*	*0.20 ± 0.011*	*36.1 ± 1.58*	*1.72 ± 0.04*	*12.2 ± 0.74*	*0.44 ± 0.010*
**60 min**	79.6 ± 1.99ND	0.99 ± 0.04*	21.6 ± 1.93**	0.27 ± 0.014***	56.0 ± 1.96***	1.38 ± 0.04***	6.2 ± 0.33***	0.53 ± 0.010**
Control	*76.4 ± 2.12*	*1.13 ± 0.04*	*31.3 ± 2.38*	*0.18 ± 0.008*	*45.5 ± 1.77*	*1.63 ± 0.04*	*10.1 ± 0.62*	*0.49 ± 0.010*
**Dose IAA**	**Density (%)**	**Skewness (a.u.)**	**Angle (°)**	**Parallelness (a.u.)**	**Density (%)**	**Skewness (a.u.)**	**Angle (°)**	**Parallelness (a.u.)**
Control[2]	*55.2 ± 1.67*	*1.28 ± 0.03*	*33.0 ± 1.35*	*0.22 ± 0.007*	*33.0 ± 1.22*	*1.81 ± 0.03*	*13.6 ± 0.54*	*0.31 ± 0.006*
**1 nM**	70.9 ± 1.45***	1.13 ± 0.03***	25.5 ± 1.28***	0.27 ± 0.010***	44.7 ± 1.14***	1.67 ± 0.03***	10.1 ± 0.37***	0.44 ± 0.007***
**10 nM**	68.0 ± 1.42***	1.19 ± 0.03*	20.6 ± 1.08***	0.24 ± 0.007ND	43.4 ± 1.08***	1.71 ± 0.02*	8.6 ± 0.31***	0.53 ± 0.006***
**100 nM**	68.2 ± 1.47***	1.19 ± 0.03*	18.2 ± 0.95***	0.30 ± 0.008***	45.6 ± 1.03***	1.71 ± 0.02*	6.7 ± 0.19***	0.58 ± 0.005***
**1 µM**	62.9 ± 1.53***	1.27 ± 0.03ND	20.1 ± 1.01***	0.29 ± 0.008***	43.6 ± 1.16***	1.66 ± 0.02***	7.8 ± 0.29***	0.51 ± 0.005***
**10 µM**	62.9 ± 1.52***	1.31 ± 0.03ND	20.6 ± 1.00***	0.31 ± 0.008***	40.7 ± 1.10***	1.80 ± 0.03ND	8.9 ± 0.28***	0.52 ± 0.006***

[1]10 nM indole-3-acetic acid (IAA) treatments at various times. Density threshold = 55.

[2]Dose series imaged at 20–30 min of treatment. Density threshold = 45.

Values are ± one standard error; a.u., arbitrary units.

Time series, N = *c.* ≥ 10 cells (Region 2) and *c.* 4–9 cells (Region 3) per root from 7 roots per treatment per timepoint.

*, $p \leq 0.05$; **, $p \leq 0.01$; ***, $p \leq 0.001$; ND = no statistical difference vs. control at timepoint, Student's t-test.

Dose series, N = *c.* 8–12 cells per region per root; at least 10 roots per treatment.

*, $p \leq 0.05$; **, $p \leq 0.01$; ***, $p \leq 0.001$; ND = no statistical difference vs. control, Student's t-test.

Methods S1 LSFM Imaging and Analysis.

The GFP-fABD2–labeled root in Video **S1** and Fig. **S11** was grown in a capillary tube as described previously (Ovečka et al., 2015), and imaged on the Bruker/Luxendo MuVi platform, a z-series halfway through the entire root every 15 min for a total of 10 h. The sample was illuminated with a 10× NA 0.3 objective (Nikon) and captured with a 20× NA 1.0 objective (Olympus) in combination with a 200 mm tube lens to produce a final magnification of 22.2×. A digital scientific complementary metal–oxide–semiconductor (sCMOS Orca Flash4 V3, Hamamatsu Photonics) captured 488-nm laser emission. Cell elongation rates were analyzed in Fiji is just ImageJ (FIJI) from a maximum projection of 140 images per timepoint; although the goal was to measure epidermal cells, for shorter cells it was not always possible to perfectly discern whether epidermal or cortical cell lengths were measured. The image stack was registered to a specific cell or set of cells, and length measurements were taken for each cell at each timepoint. Cell lengths were plotted in Excel, a line of best linear fit was fitted to each plot, and the slope of the linear trendline was used as the cell's elongation rate. To obtain elongation rates for each apparent phase of triphasic growth, growth rates were plotted for each cell when its total length was ≤ 30 μm, 30 μm ≥ 100 μm, and/or ≥ 100 μm, and included in the calculation for mean growth rate when the R^2 value of the fit line ≥ 0.85. Sample size was insufficient to calculate the growth rate of cells ≥ 100 μm because only three cells of this length met the R^2 ≥ 0.85 cutoff.

Methods S2 Genotyping Primers.

Homozygosity of *aux1-100* was confirmed using DNA primers:

**Genotype**	**Forward 5' to 3'**	**Reverse 5' to 3'**
**Wildtype WS**	GCATGCTATGTGGAAACCACAGAAG	tacCTGACGAGCGGAGGCAGATC
aux1-100	gatgcactcgaaatcagccaattttagac[1]	tacCTGACGAGCGGAGGCAGATC

[1]Forward T-DNA insertion primer from Krysan *et al.*, 1996.

Methods S3 Detailed Methods for Quantitative Analysis of Cortical Actin Array Organization.

With the exception of LSFM, all imaging was of root epidermal cells. Images were collected from the root elongation zone: root apex (i.e., root cap) to the first obviously visible root hair initiations

(RHI; end of the elongation zone/beginning of the differentiation zone). For the auxin dose and time series experiments (Figs. **3** and **S5–S6**; **Table S7**), imaging focused on Regions 2 & 3 and began at the end of the root cap (i.e., Region 1) and continued through the the first obviously visible RHI. For all other experiments, imaging included the entire root elongation zone from the root apex through first visible RHI (i.e., the beginning of the differentiation zone). Two images were collected per field of view: one to capture actin filaments in focus and one to visualize the cell side and end walls in a higher focal plane, since these are frequently clearly visible in this higher plane without staining. Each image was rotated with an image rotating macro so the longitudinal axes of the cells photographed were parallel to the horizon of the image. All micrographs were cropped and analyzed in FIJI (https://fiji.sc/). For the analysis, the overlapping images were lined up to recreate a full view of the root. In a color (RGB) version of the image stack file, cells whose side and end walls were distinguishable were identified, marked, numbered, and measured, generally choosing cells in the middle of the root to avoid including ones that might present differences in actin architecture due to differences in the cell's angle relative to the objective. On the RGB image stack, to better distinguish cells, brightness and contrast were frequently enhanced; all cropped images used for quantifying actin architecture and orientation were taken from original 8-bit files. Actin images were cropped along the entire length of every specified cell, and numbered to correspond to the specific cell from which they were cropped. Skewness and density were analyzed according to Higaki *et al.* (2010) and Henty *et al.* (2011); angle and parallelness were analyzed according to Ueda *et al.* (2010) and Cai *et al.* (2014). The size of crops must be consistent for all images in an experiment and frequently individual crops were smaller than the entire length of a cell; in such cases an actin measurement was obtained for each crop and the final scatter-plotted measurement for each actin parameter for an individual cell was taken as the mean of the measurements from that cell's particular set of crops. In Col-0 root characterization, cell size and corresponding actin architecture for more than 180 cells from at least 20 roots total were analzyed—all the cells with clearly distinguishable end walls. For effects of IAA on Col-0, cells up to 85 µm were counted as belonging to 'Region 2', cells more than 94 µm were categorized as 'Region 3', and cells falling between 85–94 µm were counted in both categories. To quantify actin architecture and orientation on a 'per-cell' basis for

the WS–aux1-100 and Col-0–aux1-22 analyses (Fig. **5** and Supporting Information Fig. **S7**, **S8**, and **S9**), the mean value from a single cell's set of crops was used as the value representing the actin measurement for that cell. For example, to fully account for all the actin in a 160 µm-long cell, 10 crops would be needed. Measurements on a per-cell basis would take the mean of the density values for those 10 crops as a single density value for that cell. In determining *aux1* response to IAA and NAA, a minimum of 125 cells (from a total of at least 9 roots) per genotype per treatment were analzyed. Relationships between actin parameters and cell dimensions were analyzed in Microsoft Excel and JMP.

Methods S4 Detailed Methods for Individual Actin Filament Dynamics.

To determine differences in actin filament behavior between shorter and longer cells, cell size was documented by taking snapshots of the entire cells from which the timelapse movies were captured. In general, movies of Region 2 cells were collected from Region 2 cells close to the root cap and movies of Region 3 cells were collected from Region 3 cells close to the end of the elongation zone (i.e., the first cell rootward of the first visible root hair initiation).

To account for differences in filament density in short and long cells, bundling, unbundling, and annealing frequencies were normalized against filament numbers in each ROI. An incident of bundling was counted as an incident in which filament fluorescence intensity increased, either from an apparent 'catch and zip' event (categorized as a 'zippering event'; these events comprise *c.* 90% of observed incidents of bundling) or, simply, a visible, unambiguous increase with a minimum three-frame persistence (3 s ≥ 10% filament lifetime) in fluorescence intensity for which 'catch and zip' was not specifically apparent (these were categorized as 'other bundling event' and account for the remaining ≈ 10% of bundling incidents). Unbundling events were counted as incidents in which a filament was visible next to a mother filament (usually 'unpeeling' over several timelapse frames) and, frequently, fluorescence intensity decreased. In cases without a visible decrease in fluorescence intensity, only events where the filament clearly 'peeled off' from the mother filament were counted as unbundling events. Incidents of annealing were counted when ends of two F-actin fragments joined together for a minimum of two frames. It was not highly unusual to see this annealing behavior join three pieces of recently severed actin

filament; if three distinguishable fragments joined to form an individual filament in the same frame, this was counted as two annealing events, one between each fragment.

When capturing timelapse movies to document individual filament changes in response to IAA within 7 min, ≈ 70 µL of either blinded solution (10 nM IAA or control) were applied directly to the microscope slide, then the root and coverslip, and imaged immediately, alternately imaging Region 2 or Region 3 first so the timepoints of each dataset would average out to 0-7 min from applying the treatment to the slide.

Video S1 Maximum projection of root epidermal cell elongation within the elongation zone over 10 h. Blue numbers label cells; elongation rates of labeled cells appear in Fig. **S11d**.

Supporting Information References

Cai C, Henty-Ridilla JL, Szymanski DB, Staiger CJ. 2014. *Arabidopsis* myosin XI: A motor rules the tracks. *Plant Physiology* **166**: 1359-1370. doi:10.1104/pp.114.244335.

Henty JL, Bledsoe SW, Khurana P, Meagher RB, Day B, Blanchoin L, Staiger CJ. 2011. *Arabidopsis* ACTIN DEPOLYMERIZING FACTOR4 modulates the stochastic dynamic behavior of actin filaments in the cortical array of epidermal cells. *Plant Cell* **23**: 3711-3726. doi:10.1105/tpc.111.090670.

Higaki T, Kutsuna N, Sano T, Kondo N, Hasezawa S. 2010b. Quantification and cluster analysis of actin cytoskeletal structures in plant cells: role of actin bundling in stomatal movement during diurnal cycles in *Arabidopsis* guard cells. Plant Journal **61**: 156-165. doi:10.1111/j.1365-313X.2009.04032.x.

Krysan, PJ, Young, JC, Tax, F, Sussman, MR. 1996. Identification of transferred DNA insertions within *Arabidopsis* genes involved in signal transduction and ion transport. *Proceedings of the National Academy of Sciences of the United States of America* **93**: 8145–8150. doi: 10.1073/pnas.93.15.8145

Ovečka M, Vaškebová L, Komis, G, Luptovčiak I, Smertenko A, Šamaj J. 2015. Preparation of plants for developmental and cellular imaging by light-sheet microscopy. *Nature Protocols* **10**:1234-1247. doi:10.1038/nprot.2015.081.

Ueda H, Yokota E, Kutsuna N, Shimada T, Tamura K, Shimmen T, Hasezawa S, Dolja VV, Hara-Nishimuraa I. 2010. Myosin-dependent endoplasmic reticulum motility and F-actin organization in plant cells. *Proceedings of the National Academy of Sciences of the United States of America* **107**: 6894-6899. doi:10.1073/pnas.0911482107.
